# EI of the Phosphotransferase System of *Escherichia coli*: Mathematical Modeling Approach to Analysis of Its Kinetic Properties

**DOI:** 10.1155/2011/579402

**Published:** 2011-03-20

**Authors:** T. A. Karelina, H. Ma, I. Goryanin, O. V. Demin

**Affiliations:** ^1^Physics Department, Moscow State University, Moscow 119991, Leninskie Gory, Russia; ^2^Institute for Systems Biology SPb, Moscow 119992, Leninskie Gory, 1/73, Russia; ^3^School of Informatics, The University of Edinburgh, Rm 2.208, Darwin Building, Kings Buildings Edinburgh EH9 3JR, UK; ^4^Okinawa Institute of Science and Technology 1919-1 Tancha, Onna, Okinawa 9040412, Japan; ^5^A. N. Belozersky Institute of Physical-Chemical Biology, Moscow 119992, Leninskie Gory, Russia

## Abstract

The mathematical model of the operation of the first enzyme of the *Escherichia coli* phosphotransferase system, EI, is proposed. Parameters of the kinetic model describing the operation of EI under different conditions are identified on the basis of a large amount of known experimental data. The verified model is employed to predict modes of operation of EI under both *in vivo* physiological conditions and *in vitro* nonphysiological conditions. The model predicts that under *in vivo* physiological conditions, the rate of phosphotransfer from EI to the second protein of the phosphotransferase system HPr by the dimer is much higher than by the monomer. A hypothesis is proposed on the basis of calculations that the transfer by a monomer plays a role in the regulation of chemotaxis. At submicromolar pyruvate concentration, the model predicts nonmonotonic dependence of the phosphotransfer rate on the substrate (PEP) concentration.

## 1. Introduction

The phosphotransferase system (PTS) of *Escherichia coli* transfers carbohydrates into the cell with simultaneous phosphorylation [1, 2]. This process operates in several steps: from the PEP, a phosphate group is transferred to EI, then to HPr, the next enzyme of the system, which, in turn, delivers a phosphate group to the enzymes EIIA and EIICB, which are specific for different carbohydrates. The glucose uptake is carried out by EIIA^Glc^ and EIICB^Glc^. The membrane-spanning enzyme EIICB^Glc^ is capable of catalyzing the transfer of a phosphate group from EIIA^Glc^ to a molecule of the relevant carbohydrate in parallel with transfer of the carbohydrate to cytoplasm. Besides the transport and phosphorylation of carbohydrates, PTS regulates metabolism of other carbohydrates, which are not PTS substrates (lactose, melibiose, etc.) [2]. In particular, the EIIA^Glc^ molecule, in addition to glucose phosphorylation, is involved in catabolite repression: in the absence of PTS-substrates, EIIA^Glc^ is mainly observed in the phosphorylated form, which activates adenylate-cyclase, and thus increases the intracellular level of c-AMP, which has an effect on the expression of a great number of genes [2]. In the presence of PTS-substrates EIIA^Glc^ is dephosphorylated. Nonphosphorylated EIIA^Glc^ takes part in a phenomenon called “inducer exclusion”. In fact, Nonphosphorylated EIIA^Glc^ is able to bind to and inhibit proteins essential in the metabolism of several carbohydrates (e.g., lactose, melibiose, maltose and glycerol) [3]. It was also shown that growth on many non-PTS carbon sources caused dephosphorylation of EIIA^Glc^ and that phosphorylation state of EIIA^Glc^ correlates with the intracellular [PEP]-[pyruvate] ratio, which is influenced by the flux through glycolysis [4]. Moreover, the transport of carbohydrate through EIICB has an impact on the activity of the transcription regulator Mlc, which controls the expression of PTS genes [3]. Therefore, PTS is a very important regulatory link in metabolism, and for understanding the mechanism of its operation, a quantitative description is required. As we did not consider any interactions of EI with other proteins, which take place in some bacteria, the model is applicable only to *E. coli, S. tiphymurium.*

The first PTS enzyme is EI, which is able to catalyze the transfer of phosphate groups from PEP to HPr (16),

(1) $$\begin{array}{c} \text{EI}+\text{PEP}\overset{\text{M}{\text{g}}^{2+}}{↔}\text{EIP}+\text{Pyr,}\\ \text{HPr}+\text{EIP}\overset{\;}{↔}\text{EI}+\text{HPrP.} \end{array}$$

This is the first enzyme of the system, and, apparently, in many respects its operation determines the ratio between the concentrations of phosphorylated and Nonphosphorylated forms of the other PTS enzymes. EI is known to exist in monomeric and dimeric form [5]. The C-terminal domain of the monomer bears the PEP-binding site and is important for dimerization, while the N-terminal domain contains a site of phosphorylation (His-189) and participates in the transfer of phosphate to HPr [6]. Only a dimer is able to accept a phosphate group from PEP, while the Nonphosphorylated monomer is involved in regulation of chemotaxis [7]. Thus, this enzyme is an important regulatory link in cell activity.

Despite a substantial number of studies of this enzyme [5, 6, 8–17], the mechanism of its operation remains obscure. It is known that the presence of PEP and magnesium ions in the medium promotes dimerization of phosphorylated EI subunits and considerably shifts equilibrium towards the formation of dimers [8, 10]. The dimerization stage is one of the slowest steps in the enzyme operation; it is most likely a limiting one and, thus, determines the rate of its operation. It has not yet been clearly identified what is the mechanism of phosphate group transfer from EI to HPr, the next component of PTS: it is unknown if a dimer or a monomer performs the transfer. As a matter of fact, the literature provides evidence that although PEP promotes dimerization, phosphorylation of subunits itself weakens their binding [8], and in this connection, a hypothesis about a cyclic mechanism of EI functioning has been proposed [8]: dimerization-phosphorylation of the dimer dissociation of the dimer to phosphorylated monomers—a transfer of phosphate from monomer to HPr—dimerization. At the same time, some data indicate that phosphorylation stabilizes a dimer [11], which rather supports the hypothesis involving transfer by the dimer. Moreover, it is known that the N-terminal domain of a monomer can abstract a phosphate group from phosphorylated HPr (HPrP); that is, interaction between HPr and the monomer of EI must not be excluded [9]. This implies that in developing a kinetic model, it is necessary to take into account both mechanisms of transfer of a phosphate group.

A wide range of different kinetic evidence has been found for the operation and regulation of the EI enzyme. In several studies [5, 6, 15–18], the time dependences of the concentration of the phosphorylated protein, the dependences of the initial phosphotransfer rate on substrate concentrations, and so forth, have been measured. However, there exists no complete model including dimerization of the enzyme, interaction with substrates and products, describing simultaneously all these experimental data. Only such a model may allow understanding the mechanism of operation of this enzyme. In this study, a kinetic model of EI has been developed, describing satisfactorily a large set of experimental data, and a series of conclusions on its operation has been made. In particular, based on the analysis of constants, cooperative properties of the enzyme have been found, and the dependence of the rate of enzyme operation on the concentration of substrate and product (PEP and Pyr) has been analyzed. The transfer of phosphate to HPr has been shown to be carried out both by dimeric and monomeric forms of EI, and a feasible physiological role of such transfer by a monomer has been found.

## 2. Models

### 2.1. Known Experimental Data and Hypotheses Used for the Model Development

To understand the operating mechanism of Enzyme I, all available data from literature were collected. These experimental facts were used to reconstruct the catalytic cycle of EI.

1. EI can exist both as a monomer and as a dimer [5].
2. Binding of the substrate (PEP) is a very rapid process judging from the immediate changes in the fluorescence intensity of Trp groups during fluorescence spectroscopy studies, as noted by authors of a previous study [13].
3. PEP binding accelerates dimerization and shifts the balance towards the formation of a dimer [8, 10].
4. The process of dimerization is sensitive to temperature and pH [8, 14].
5. Only a dimer can accept a phosphate group from PEP [5].
6. It is unknown whether a dimer or a monomer transfers phosphate group to HPr, but it was shown that phosphorylated HPr can phosphorylate a monomer EI [9].

To simplify the description of enzyme operation, a number of assumptions have been made.

1. Since the substrates are bound to the C-terminal domain of the EI subunit and phosphorylation of HPr is carried out by the N-terminal domain [6] and Pyr bears no charged phosphate groups, which could affect some processes, it was assumed that Pyr binding to the enzyme has no effect on the rates of phosphate transfer from EI to HPr (the list of the rates is represented in Supplement 2, of Supplementary Material, which is available online at doi:10.1155/2011/579402).
2. We assumed that temperature influence on some processes similarly, and thus some kinetic constants similarly depend on temperature. So, some processes have equal enthalpies or equal activation energies (see the list of subsequent enthalpies and activation energies in Supplement 2 (S.29.1-2)).
3. Substrate (PEP) and product (Pyr) binding occurs much faster than the reactions of dimerization and phosphorylation (i.e., they are in quasiequilibrium).
4. Since literature provides the data that phosphorylation of HPr occurs very quickly (equilibrium is reached within 1 s [6], while in the other processes reaching equilibrium takes much longer), the process of formation of the EI-HPr complex has not been taken into account.

There are no experimental data confirming these assumptions, but they seemed reasonable and were used to decrease the number of unknown kinetic parameters.

### 2.2. The Catalytic Cycle

**Figure 1:**
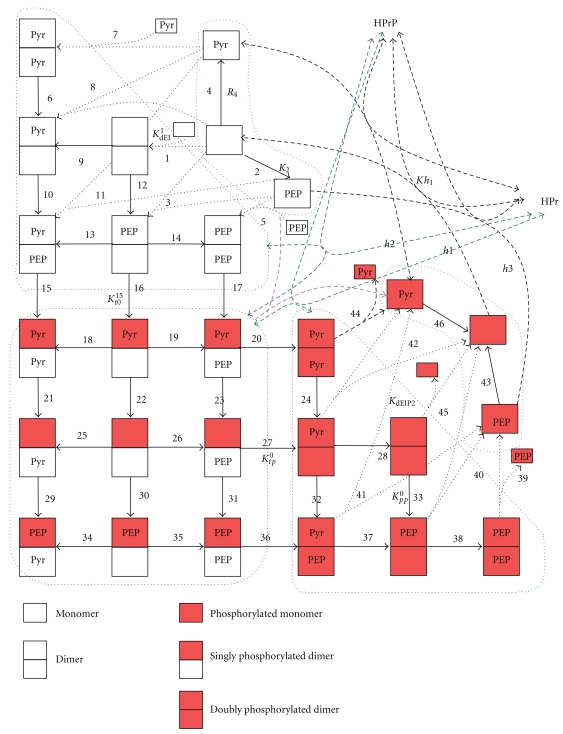
Catalytic cycle of Enzyme I of PTS. Designations for dimer, monomer, and phosphorylated subunits are shown on the picture. Elementary processes are denoted by the arrows with numbers: processes of PEP and Pyr binding and EI phosphorylation are denoted by solid lines, dimerization (dissociation) processes are denoted by the dotted lines, and processes of the phosphate transfer to HPr denoted by the dashed lines with numbers and letter “h”. For example, there are also designations of the equilibrium constants for different types of the reactions given (description of the types see in Supplement 1. Free PEP and Pyr are not shown, numbers of the reactions of PEP, and Pyr binding are given in the Supplement 1). Lilac dashed lines: sum of the processes of singly phosphorylated dimer dissociation (Figure 2), green dashed lines: sum of the processes of the HPr phosphorylation by the doubly phosphorylated monomer (Figure 3(a)), blue dashed lines: sum of the processes of the HPr phosphorylation by the singly phosphorylated monomer (Figure 3(b)).

**Figure 2:**
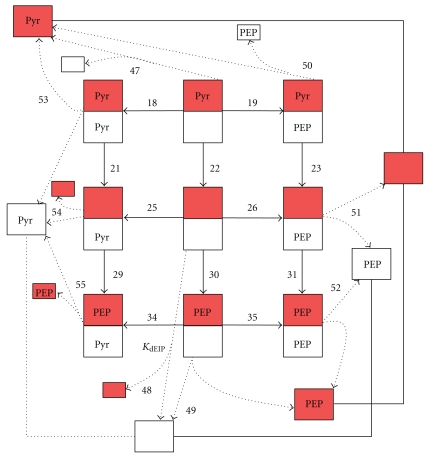
Dissociation of the singly phosphorylated dimer to monomers. Designations are as Figure 1 and as in the text. Dimer dissociation is shown by the dotted lines.

**Figure 3:**
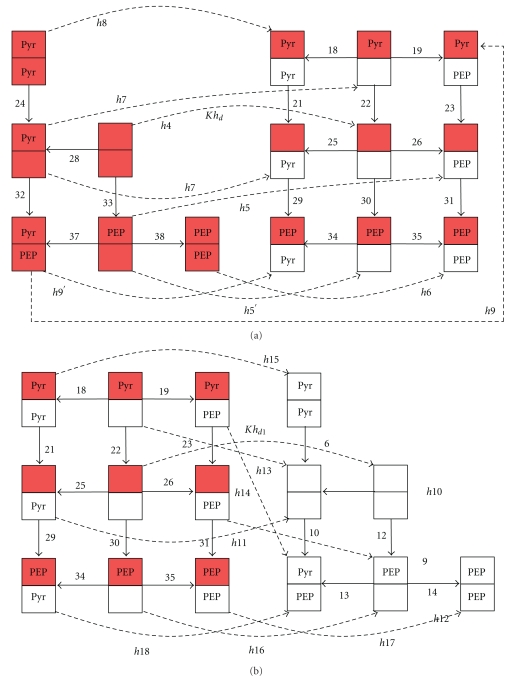
Phosphorylation of the HPr by the form of EI dimer. (a) doubly-phosphorylated dimer; (b) singly-phosphorylated dimer. Designations are as Figure 1 and in the text.

Consistent with these experimentally proved data and assumptions, a catalytical cycle of the enzyme has been developed (see Figures 1–3). As has been mentioned in the introduction, the site of PEP binding and the site of phosphorylation are situated on different domains, so PEP and Pyr supposedly can bind to the phosphorylated form of EI. Experiments allow us to measure only the apparent kinetic parameters. At the same time, experimental data show that there are many regulatory interactions in this system. As the aim of our work was the description of all available experimental data, we should take into account all these processes. In the proposed catalytic cycle, interaction of all the substrates and products with various forms of the enzyme is considered. For the aims of simplification of the model and decreasing number of parameters, we did not considered particular stages of conformational changes like, for example, conformational change of EI after phosphorylation by PEP [19]. Phosphorylation and conformational change that His-domain undergoes after it are joined in general stages of EI-phosphorylation (reaction numbers 15, 16, 17, 20, 27, and 35). It was assumed that both a dimer and a monomer can transfer a phosphate group to HPr. All the reactions shown in Figures 1–3 are reversible. The rate constant of the dimerization was hardly affected by Mg^2+^ (<20%) [10] at the concentration range used in all experiments (2–10 mM), Mg^2+^ always was added to the buffer in the experiments that we used for parameter identification, and, therefore, Mg^2+^ was not considered as a variable in the catalytic cycle. According to the above-noted experimental data, Figure 1 shows that both dimers and monomers exist, reversible dimerization of Nonphosphorylated (reaction numbers 1, 3, 5, 7, 8, and 11) and phosphorylated monomers (reaction numbers 39, 40, 41, 42, 44, and 45) is possible and only a dimer can accept phosphate from PEP (reaction numbers 15–17, 20, 27, and 36). Figure 1 also presents different enzymatic states: a dimer, a monomer, a phosphorylated monomer, a singly phosphorylated, and a doubly phosphorylated dimer, each of which can exist with or without bound PEP or Pyr (description and designations of all the states are given in the legend to Figure 1). Thus, one of the possible processes of phosphate transfer from PEP to HPr may be as follows: Nonphosphorylated monomers of EI dimerize (reaction number 1), PEP binds to a dimer (reaction number 12), the subunit with bound PEP is phosphorylated (reaction number 16), Pyr dissociates from the phosphorylated dimer (reaction number 22), PEP binds to the Nonphosphorylated subunit of the dimer (reaction number 26), this subunit is phosphorylated (reaction number 27), Pyr dissociates from the doubly phosphorylated dimer (reaction number 28), doubly phosphorylated dimer dissociates to monomers (reaction k_45_), and the monomer transfers phosphate to HPr (reaction number h2). The other processes may differ in the enzymatic state during the transfer of phosphate to HPr (by the dimer (see Figure 3) or the monomer form of EI) and in the availability of the substrates bound to the subunits (if, e.g., Pyr has not dissociated or PEP is bound to an already phosphorylated subunit of EI). A part of the designations in Figure 1 corresponds to the sum of the states. For example, if only one PEP molecule dissociates from the dimer with two bound PEP molecules (reaction 14), then this reaction can take place on either of the subunits with equal probability, and, in the end, the final states will be indistinguishable. If the constant of PEP dissociation from one of the subunits of the dimer in this state equals ${\tilde{K}}_{14}^{}$, then the corresponding apparent equilibrium constant of the process of PEP dissociation from the dimer with two bound PEP molecules is taken to be $K_{14}^{}=2\cdot {\tilde{K}}_{14}^{}$ (statistical weight factor 2 appears). The statistical weight factors are listed in Table S.1 in Supplement 5 next to the parameters. Expressions are given only for the independent constants; the other constants are expressed in terms of the independent constants by using the ratios of detailed balance (Supplement 2). Although Figure 1 shows all the EI states described in the model, it was not feasible to show also in this figure all the possible transfers between the states. However, in Figures 2 and 3 all the transitions between the states are to be found: the dissociation of the singly phosphorylated dimer to the monomers and the transfer of the phosphate group to HPr by the singly phosphorylated dimer and the doubly phosphorylated dimer.

### 2.3. Description of the Model

**Figure 4:**
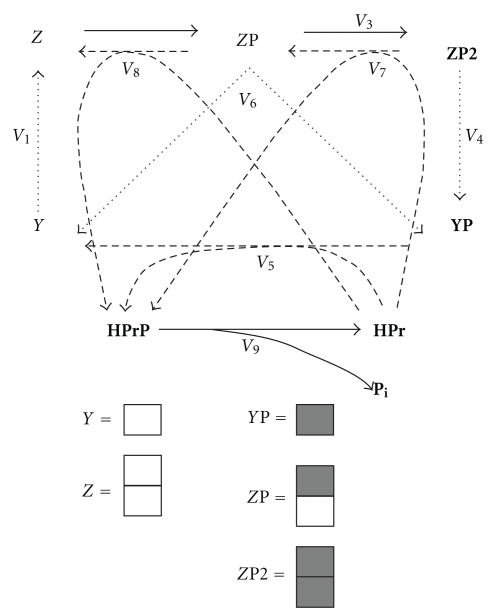
Catalytic cycle of Enzyme I in reduced form. Designations *Y*: the sum of the concentrations of Nonphosphorylated monomer forms; *YP*: the sum of the concentrations of the phosphorylated monomer forms; *Z*: the sum of the concentrations of Nonphosphorylated dimer forms; *Z*P: the sum of the concentrations of singly-phosphorylated dimer forms; *Z*P2: the sum of the concentrations of the doubly-phosphorylated dimer forms. New rates correspond to the sums of the elementary processes rates (see explanation in the main text and Supplement). Dashed lines: phosphotransfer to HPr, dotted lines: dissociation (dimerization) reactions, other reactions are shown by the solid lines.

 With the help of the assumption (3), given above, the catalytic cycle of the enzyme EI was reduced (Figure 4). In the reduced catalytic cycle, we substituted the previous variables (the enzymatic states in Figure 1) for new ones which represented the sums of the concentrations of the EI forms (Supplement 1). Figure 4 shows the new variables, which have the following physical meaning: *Y* is the total concentration of the states of the Nonphosphorylated monomer, *Z* is the total concentration of the states of the Nonphosphorylated dimer, *Z*P is the total concentration of the states of the singly phosphorylated dimer, *Z*P2 is the total concentration of the states of the doubly phosphorylated dimer and *YP* is the total concentration of the states of the phosphorylated monomer. All the transitions between these new states are described by rate equations which are the sums of the rates of elementary reactions of the nonreduced catalytic cycle (Supplement 1). The concentration of every enzyme state given in Figure 1 can be expressed via the new variables shown on Figure 4. Supplement 1 gives, for example, the detailed derivations of the expressions for EI^PEP^ (concentration of the Nonphosphorylated monomers with bound PEP) and EI^Pyr^ (concentration of the Nonphosphorylated monomers with bound Pyr) through the new variables. The expressions for the other states of the complete catalytical cycle can be obtained in a similar way. Changes in the concentrations of substrate PEP and product Pyr are described by the laws of conservation ((9), (10)): in the experiments the number of the phosphate groups and the number of “carbonic bases” (PEP + Pyr) are fixed (see Supplement 1, formulae S.25.1-25.2).

In addition, in the model, we also take into account the fact that HPrP hydrolyses (the concentration of phosphorylated protein decreases to 15% of the initial value in 30 minutes [17]): the rate of hydrolysis is designated as *V*_9_. Hence, Model I, describing the reduced catalytical cycle and taking into account that concentrations of PEP, Pyr and HPr are the variables, consists of 6 differential ((2)–(7)) and 4 algebraic ((8)–(11)) equations

(2) $$\begin{array}{cc} & \frac{dY}{dt}=-2\cdot V_{1}+V_{5}+V_{6}, \end{array}$$

(3) $$\begin{array}{cc} & \frac{dZ}{dt}=V_{1}-V_{2}+V_{8}, \end{array}$$

(4) $$\begin{array}{cc} & \frac{dZ\text{P}}{dt}=V_{2}-V_{3}-V_{8}+V_{7}-V_{6}, \end{array}$$

(5) $$\begin{array}{cc} & \frac{dY\text{P}}{dt}=2\cdot V_{4}-V_{5}+V_{6}, \end{array}$$

(6) $$\begin{array}{cc} & \frac{d\text{HPrP}}{dt}=V_{5}+V_{7}+V_{8}-V_{9}, \end{array}$$

(7) $$\begin{array}{cc} & \frac{d{\text{P}}_{\text{i}}}{dt}=V_{9} \end{array},$$

(8) $$\begin{array}{cc} & Y+2\cdot Z+2\cdot Z\text{P}+2\cdot Z\text{P}2+Y\text{P}={\text{EI}}_{\text{total}}, \end{array}$$

(9) $$\begin{array}{cc} & \text{PEP}+\text{PTSP}+\text{EI}\circ \text{PEP}+{\text{P}}_{\text{i}}=\text{PEP}\left(0\right)+\text{PTSP}\left(0\right), \end{array}$$

(10) $$\begin{array}{cc} & \text{Pyr}=\text{Pyr}\left(0\right)+\left(\text{PTSP}-\text{PTSP}\left(0\right)\right)-\text{EI}\circ \text{Pyr}, \end{array}$$

(11) $$\begin{array}{cc} & \text{HPrP}+\text{HPr}={\text{HPr}}_{\text{total}}. \end{array}$$

Here, EI_total_, HPr_total_ are the pools of EI monomers and HPr protein, respectively, PTSP is the sum of the concentrations of the phosphorylated states of EI and HPr proteins, so that

(12) PTSP=ZP+2·ZP2+YP+HPrP.

PEP(0), Pyr(0), PTSP(0) are the initial concentrations of PEP, Pyr, PTSP, respectively; P_i_ is inorganic phosphate; EI∘PEP, EI∘Pyr are the total concentrations of the forms of the enzyme to which PEP and Pyr are bound, respectively, (the expressions through the variables of the reduced model are given in Supplement 1, S.21.1-2); designations of the variables and the reaction rates correspond to Figure 4. A differential equation for *Z*P2 is not given because this concentration was calculated from (8).

To describe the data on the initial rates of HPr phosphorylation, it is convenient to use the simplified model (Model II) in which changes of the concentrations of PEP, Pyr and HPr are not taken into account, and concentrations of HPrP and P_i_ are fixed to zero. This model consists of 4 differential equations (2)–(5) and one algebraic (8) equation; that is, it represents a subsystem of Model I.

### 2.4. Dependence on Temperature and pH

 As the experimental data we wanted to use for verification of the models were not measured at one and the same value for pH and temperature, our model should be able to account for the influence of these factors on the described processes. Since the values of pH and the temperature are known to affect the kinetic parameters, (e.g., [14]), the model has to take into account the dependence of the constants on temperature and pH. The dependences of the equilibrium constants on temperature were described according to the van't-Hoff equation:

(13) $$\frac{K_{i}^{}\left(T\right)}{K_{i}^{}\left(T_{0}\right)}=\exp\;\;\left(\frac{\Delta H_{i}\cdot \left(T_{0}-T\right)}{R\cdot T\cdot T_{0}}\right).$$

Here, *K*_*i*_(*T*) is the equilibrium constant of the *i*th elementary reaction at the temperature value *T*, *K*_*i*_(*T*_0_)- is the equilibrium constant of the *i*th elementary reaction at the temperature value *T*_0_, Δ*H*_*i*_ is the reaction enthalpy and *R* is the universal gas constant.

The dependences of the rate constants on temperature were described according to the Arrhenius equation

(14) $$\frac{k_{i}\left(T\right)}{k_{i}\left(T_{0}\right)}=\exp\;\left(\frac{E_{ai}\cdot \left(T_{0}-T\right)}{R\cdot T\cdot T_{0}}\right).$$

Here, *k*_*i*_(*T*) is the rate constant of the *i*th elementary reaction at the temperature value *T*, *k*_*i*_(*T*_0_) is the rate constant of the *i*th elementary reaction at the temperature value *T*_0_, *E*_*ai*_ is the activation energy.

The dependence on the pH value is calculated by the method proposed by Cornish-Bowden [20]. It is supposed (assumption (0)) that an enzyme (or, more precisely, its monomer), is a dibasic acid, and only the singly protonated form is catalytically active. The detailed description of calculations and formulae are given in Supplementary 3. 

The rate of HPrP hydrolysis was considered to be independent of temperature and pH.

The Models I and II described above have different numbers of variables. These models describe different experiments. However, one set of the parameters was used by us for all the calculations (63 equilibrium constants, 46 rate constants, 12 proton dissociation constants, 75 enthalpies, and 46 activation energies).

For developing a model and making the calculations, the DBSolve 7 program was used [21, 22].

### 2.5. Description of the Parameters of a Model and Experimental Data Used for Their Identification

According to the ratios of the detailed balance, 32 equilibrium constants are expressed through the other ones, hence, the number of unknown equilibrium constants was reduced from 63 to 31. In addition, according to the assumptions (1) and (2) of Section 2.1, we decided to equate some of the parameters with each other (see Supplementary 2). Thus, 137 independent parameters remained, among them 31 equilibrium constants, 12 proton dissociation constants, 37 rate constants, 31 enthalpies, and 25 activation energies. The number of unknown parameters was too large for “manual” analysis. To select the values of the parameters we used the algorithm of fitting in the program DBSolve. As a criterion of fitness, the following function was used:

(15) $$f\left(k_{j},\;K_{j}\right)=\overset{n}{\underset{i}{\sum }}{\left(v_{i}-{\overset{®}{v}}_{i}\right)}^{2}.$$

Here, *n* is the total number of experimental points, ${\overset{®}{v}}_{i}$ is the experimentally measured value of the variable or reaction rate, *v*_*i*_ is the value of the variable or reaction rate calculated based on the model at a point corresponding to the experimental ones. To estimate values of unknown parameters, the error of the model (*f*) has been minimized. This procedure was performed in the DBSolve 7 package using the Hook-Jeeves method [23]. Sensitivity of the error to the parameters we evaluated using range of sensitivity, values of parameter giving twice increase of *f*.

With such a number of parameters, it is rather difficult to determine them unambiguously, and, therefore, we tried to fit them to the maximally possible number of experimental data values: 175 experimental points at pH values from 6.5 to 8 and temperature values from 23°C to 37°C [5, 6, 17, 18]. The fitting procedure was performed in three steps. First, the parameters for phosphotransfer from PEP to EI were determined by fitting them to the data for EI phosphorylation without HPr. Then, these parameters were fixed, and other parameters were determined by fitting to the experimental data on HPr phosphorylation. In the third step, all the parameters were fitted together to all kinds of the data. Third step was conducted for different kinds of models of phosphotransfer to HPr: (i) phosphotransfer by the monomer, (ii) phosphotransfer by the doubly phosphorylated dimer, (iii) phosphotransfer both by doubly and by singly phosphorylated dimmer, and (iv) phosphotransfer by all forms of EI. We tried to start fitting from different initial values of the parameters. The resulting set of parameters was the only one that allowed satisfactory description of the experimental data. Of course, there is no way to check if this set is unique. However, it is validated by the fact that some values of parameters are close to those experimentally observed (see Table 2) and that it allows the description of some experimental data that were not fitted (see below). Upon fitting, some of the enthalpies and activation energies appeared to be close to zero. We have fixed these parameters to zero; that is, we have assumed that the corresponding equilibrium constants and rate constants are not dependent on temperature. Some of the rate constants were also close to zero, so, in the calculations, we fixed them to zero. Hence, 90 independent parameters with values different from zero remained. For some of the parameters fitted values have been compared with experimentally observed values from other references, and ranges of sensitivity have been calculated for these parameters. 

The data from [11] contradict the data from reference [8]. As the [8] and other papers of this research group contain a lot of experimental kinetic results, we have chosen these results for fitting. Results from [11] were not taken into account.

## 3. Results and Discussion

### 3.1. Comparison of Different Models

After fitting of different models, we compared them with the help of Akaike information criterion (AIC) [24]

(16) $$\text{AIC}=\ln\;\frac{s^{2}}{n}+2\cdot q,$$

where

(17) $$s^{2}={\overset{n}{\underset{i}{\sum }}\left(\frac{v_{i}-{\overset{®}{v}}_{i}}{v_{i}}\right)}^{2},$$

and *q* is the number of parameters. The results of comparison are given in Table 1.

It can be seen that model with phosphotransfer by different forms of EI provides the minimal value of AIC, thus it have been chosen for description of EI operation.

Since we have fitted a lot of data, we have deposited most of the results in the Supplemental Material (Supplement 4). Only the most interesting kinetic curves are presented in the main text. The values of the parameters obtained are given in Table S.1 of Supplement 5.

### 3.2. EI Phosphorylation

**Figure 5:**
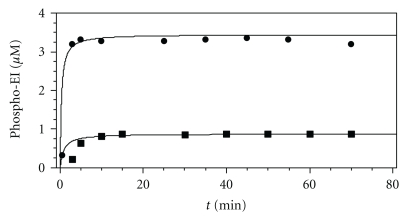
Fitted experimental data from [16]. Changing of the concentration of the phosphorylated EI with time (without HPr); EI concentrations: (■): 0.06 mg/mL; (●): 0.23 mg/mL; 20-fold molar excess of PEP was used, T = 23°C, pH = 6.5.

Figure 5 and Figure i of the Supplementary 4 show the results of fitting the model to the experimental data. Figure 5 shows the results of the calculations using Model I (in the absence of HPr) with previously published experimental data obtained *in vitro* [17]. We studied the kinetics of enzyme I phosphorylation. In the published work [17], the quantity of the phosphorylated monomers of the protein EI was measured at different time points after mixing PEP and EI. The analogous experimental and theoretical curves are given in Figure i(a) in the Supplement 4. In addition, we have described experiments in which the quantity of phosphorylated EI was measured at various initial concentrations of EI and PEP upon reaching equilibrium at different values of pH (Figure i(b, c), Supplement 4), as well as at various initial values of Pyr and invariant initial concentrations of EI and PEP (Figure i(d) of the Supplement 4). The degree of enzyme phosphorylation is described satisfactorily.

### 3.3. HPr Phosphorylation

**Figure 6:**
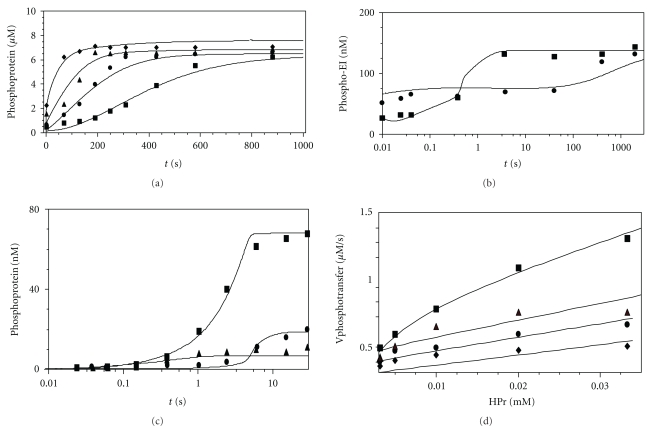
Fitted experimental data on phosphorylation of the both EI and HPr. (a) experimental data from [18]: time dependences of the concentration of the phosphorylated protein (HPrP + EIP): *T* = 37°C, pH = 7.4. Initial concentrations of components: PEP, 160 *μ*M, HPr, 24.4 *μ*M, EI (Nonphosphorylated monomers), (■) 157 nM, (●) 312.5 nM, (▲) 729 nM, (♦) 1.57 *μ*M; (b),(c) Experimental data from [6]: time dependences of the concentrations of the phosphorylated EI and HPr; *T* = 25°C, pH = 6.5, Initial concentrations of the components: (b) EI (Nonphosphorylated monomers): 140 nM, PEP: 44 mkM, HPr (when present): 1.85 mkM; designations of data points: (●) phospho-EI in the absence of the HPr, (■) phospho-EI in presence of the HPr; (c) initial concentrations: EI (Nonphosphorylated monomers): 32.8 nM, PEP, 390 nM, HPr (when present): 66 nM HPr, (●) phospho-EI in the absence of the HPr, (■) phospho-EI in presence of the HPr, (▲) HPrP. (d) Experimental data from [5]: dependence of the initial HPr phosphorylation rate on the HPr concentration; *T* = 25°C, pH = 7.2, EI (total), 1.3 *μ*M, PEP concentrations (mM): (■)—2, (●)—0.215, (▲)—0.115, (♦)—0.0615.

Figure 6 shows the results of fitting obtained with Model I (Figures 6(a), 6(b), and 6(c)) and II (Figure 6(d)) and the experimental data on the kinetics of HPr phosphorylation, obtained from several published works [5, 6, 18]. In general, the model describes the experimental data accurately.  Figure 6(a) indicates that the model describes the time course of HPr phosphorylation [5, 18] registered at various concentrations of EI. *In vitro*, as well as *in silico*, the equilibrium concentration of HPrP depends only weakly on the EI concentration, whereas the time for reaching equilibrium is reduced with increasing concentration of EI. Figures 6(b) and 6(c) shows the experimental data from the reference [6] and the fitting results obtained with Model I describing the phosphorylation of EI and HPr at different initial concentrations of the components EI, HPr and PEP.  Figure 6(b) shows the dependence of the concentration of phosphorylated EI on time in the absence or presence of HPr [6].  Figure 6(c) gives the dependence of the concentration of phosphorylated EI on time without HPr, as well as the dependence of the concentration of phosphorylated EI and HPr on time in the presence of HPr at low concentrations of the components EI, HPr, and PEP [6]. The results describing other kinetic curves from the reference [6] are given in Figure ii of the Supplement 4 ((a): EI phosphorylation with HPrP in the absence of PEP, (b): HPr phosphorylation in the same experiment as in Figure 6(b) in the main text). It is observed that with different initial concentrations of the components the model describes efficiently both the equilibrium concentrations of the products (phosphorylated proteins) and the kinetics of the transition to equilibrium. Figure 6(d) shows the dependences of the initial rate of HPr phosphorylation on the HPr concentration at various concentrations of PEP [5] calculated using Model II. With increasing concentration of PEP, the curves of the rate dependences on HPr are elevated. The curves of the dependences of the initial rate of HPr phosphorylation on PEP concentration at different initial concentrations of HPr are given in Figure iii of the Supplement 4. These experimental points are also described successfully with the model. These data have also been fitted using the simplified model (Model II).

Note that all the experimental data have been described using one set of parameters, and these parameter values provide their satisfactory description.

After fitting, we have checked whether the model could describe data which were not fitted. Figure iv of the Supplement 4 shows that our model describes rather well the experimental data of [17] on the EI phosphorylation at the pH values of 7.0 and 8.0. Besides, for fitting of the data on the initial phosphotransfer rates (Figure 6(d) and Figure iii of the Supplement 4), we used only 19 experimental points out of 40 presented in [5]: we took 2-3 points from each curve of the dependence of the reaction rate (1) on the substrates HPr and PEP. Nevertheless, the model satisfactorily describes all the experimental points (Figure 6(d)). Thus, the parameter values found with the help of fitting to the 175 points are suitable for the description of the other 31 experimental points. This may serve as verification of the predictive power of the model.

### 3.4. Analysis of the Kinetic Properties

Upon description of all the experimental data, we aimed to analyze which additional properties of the Enzyme I the proposed model would describe and predict employing the single set of the parameter values found.

#### 3.4.1. Physiological Role of the Different Mechanisms in the Transfer of the Phosphate Group to HPr

**Figure 7:**
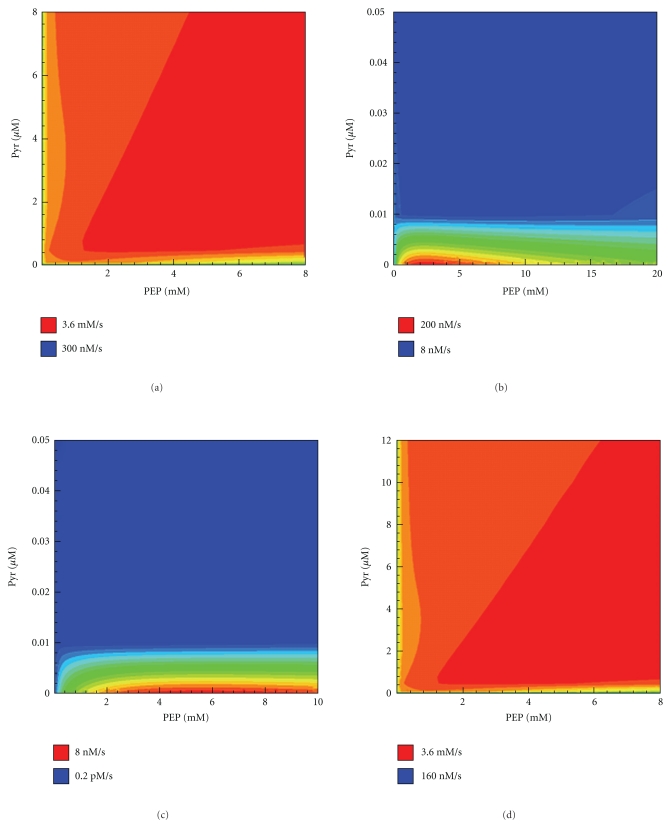
Dependence of the steady state HPr phosphorylation rate on the PEP and Pyr concentrations (contour maps) calculated by the Model II. (a) total stationary rate of HPr phosphorylation. Also shown contributions (to the total HPr phosphorylation rate) of rates of the HPr phosphorylation: (b) by the monomer; (c) by the doubly phosphorylated dimer; (d) by the singly phosphorylated dimmer Concentrations of the enzymes are close to physiological ones (taken from [25]), which are: EI = 5 *μ*M, HPr = 40 *μ*M.

One of the most interesting issues in the catalytic cycle of EI is the significance of the existence of two different ways of transfer of the phosphate group to HPr. First, we tried to describe the experimental data with the model of “a cyclic mechanism”, which has been proposed by Chauvin et al. [8]. In this model, we supposed that for the transfer of the phosphate group to HPr the doubly phosphorylated dimer should dissociate to form monomers. However, the cyclic model has failed; in the experiments described in [6], the system reached equilibrium much faster than it did in our model at the dimerization rate constant value close to the one measured in [10]. Obviously, it takes much more time to pass through a complete cycle of the dimerization-dissociation [6]. Upon taking into account the transfer of phosphate to HPr not only by a monomer but also by a dimer we managed to describe this data (Figures 6(b) and 6(c), e.g.). Thus, with a phosphorylated dimer as the active species, a rapid transfer of the phosphate group to HPr was established. Further analysis has shown that transfer by singly phosphorylated dimer constitutes the main contribution to the phosphate transfer flux to HPr. In the absence of Pyr, about 2/3 of the flux is realized by the singly phosphorylated dimer, and about 1/3 is transferred by the monomer. Figure 5 shows in the form of the contour plot the dependences of the rates of HPr phosphorylation by various forms of EI on the substrate (PEP) and the product (Pyr) concentrations at the physiological concentrations of EI and HPr [26]. When the Pyr concentration equals 0.01 mM and higher, the contribution of the transfer reactions made by the monomer into the total phosphate transfer flux becomes still lower: the transfer is established almost completely by the singly phosphorylated dimer. The rates of phosphate transfer to HPr by the monomer (Figure 7(b)) and the doubly phosphorylated dimer (Figure 7(c)) decrease rapidly with increasing concentration of Pyr. In this case, the total rate of phosphate transfer to HPr (Figure 7(a)) coincides almost completely with the rate of phosphate transfer by singly phosphorylated dimer (Figure 7(d)). Figures show the small region for the Pyr concentrations in the submicromolar range, because only in this region can any significant changes be seen (Figures 7(b) and 7(c)). As has been calculated, in the range of millimolar Pyr concentrations the rate of phosphotransfer by *Y*P and *Z*P2 would have been very low, corresponding to the blue field, so there will not be any qualitative changes. Thus, singly phosphorylated dimer makes the main contribution to the rate of phosphate transfer to HPr in the presence of Pyr even in low concentrations (Figure 7(d)). 

Although physiological concentrations of PEP and Pyr are usually in the millimolar range, it has been shown that in some cases they could reach micromolar values. In fact, physiological concentrations of PEP and Pyr can decrease to 0.1-0.2 mM under conditions of carbon limitation after addition of a glucose pulse, as has been demonstrated in [27]. Other experiments on the addition of glucose to glucose-starved cells are in agreement with these results: in [28], it was shown that PEP concentration had fallen to undetectable values, less than 0.05 mM, and in [29], it was shown that PEP concentration after addition of glucose had fallen to 22 *μ*M, and Pyr concentration had fallen to about 16 *μ*M. So, studying the behaviour of the PTS in conditions of low PEP and Pyr concentration may have some physiological significance. 

**Figure 8:**
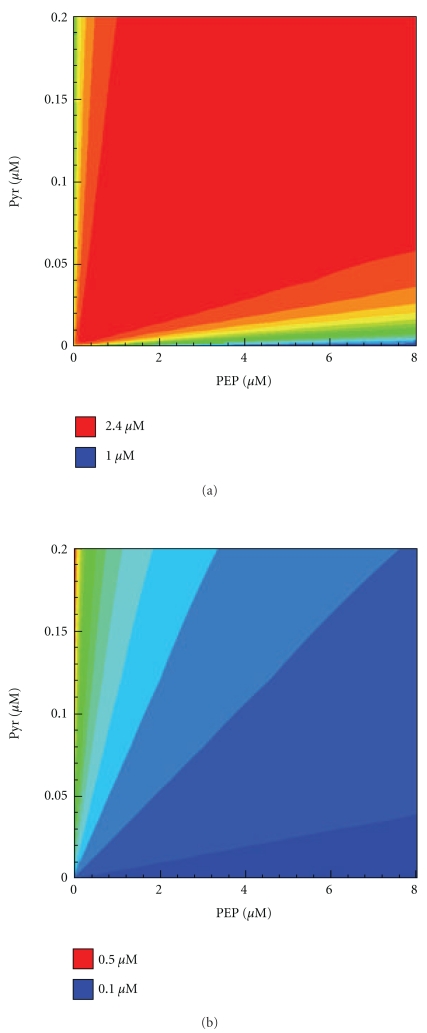
Dependence of the steady state Nonphosphorylated monomer concentration on the PEP and Pyr concentrations as calculated by the Model II for different mechanisms of HPr phosphorylation. (a) including phosphate transfer to HPr by the monomer; (b) without phosphate transfer to HPr by the monomer (*kh*_1_ = *kh*_2_ = *kh*_3_ = 0). Concentrations of the enzymes are as given in the legend of Figure 5.

To shed light on the physiological role of phosphate transfer by the monomer, we analyzed how the presence or absence of this pathway of transfer affected the state of the system. Figures 8(a) and 8(b) show the dependences of the *Y* concentration on the PEP and Pyr concentrations for both cases mentioned above. The red field corresponds to relatively high concentrations of Nonphosphorylated monomers and consequently to activation of chemotaxis. If the rate constants of phosphate transfer by the monomer were set to zero, very low concentrations of the Nonphosphorylated monomers were obtained (Figure 8(b)). The Nonphosphorylated monomer is known to play an important role in the regulation of chemotaxis by activating this process [7]. The transfer of the phosphate group by *Y*P regenerates free monomers and, hence, causes activation of chemotaxis. If this pathway is absent, then we should observe very low concentrations of the Nonphosphorylated monomers; that is, even in the presence of PTS-substrates in the medium, the cell would not follow the gradient of substrates. It was shown in [7] that *in vitro *inhibition of CheA by *Y* begins at a *Y* concentration of a 3-fold excess over CheA. However, *in vivo* concentrations of EI and CheA are comparable (CheA = 2–4 *μ*M, EI = 2–6 *μ*M) and a *Y* concentration of 2.4 *μ*M (Figure 8(a)) may be sufficient to regulate chemotaxis. Figure 8(a) shows that conditions are favourable (chemotaxis) if the Pyr/PEP ratio is in the 0.006–0.03 range. If Pyr concentration is much lower than PEP concentration or *vice versa*, if PEP is much lower than Pyr, there will be virtually no Nonphosphorylated monomers present in the cell and chemotaxis would not be activated. In the absence of transfer by the monomer (Figure 8(b)), the Nonphosphorylated monomer concentration is higher for low PEP concentrations, and, hence, a cell would activate chemotaxis without reason. Another way for regeneration of *Y* could be dissociation of Nonphosphorylated dimer. This possibility was checked by preventing *Z* dissociation *in silico*. Interestingly, this did not affect either the level of Nonphosphorylated monomers *Y* nor the rate of phosphotransfer to HPr. This means that the main path of regeneration of *Y* is the transfer of the phosphate group from *Y*P to HPr. In conclusion, both mechanisms of transfer of the phosphate group play a significant role in the cell.

#### 3.4.2. Studying the Obtained Values of the Parameters

The obtained values of all the parameters are given in Table S.1 of Supplement 5. The analysis of the constants obtained with fitting have shown that some enthalpies and activation energies describing the temperature dependence of the kinetic parameters have been close to zero. This, obviously, means that these processes do not depend on the temperature. 

As we have determined efficient values of the constants, we have listed simple statistical multipliers in Table S.1 of Supplement 5 against the names of the constants. Thus, for example, we have determined an efficient constant *K*_14_, but, as it is equal to $2\cdot {\tilde{K}}_{14}^{}$ (see Section 2.2), a statistical weight factor of 2 is used.

The values of the dissociation constants of unphosphorylated dimer were close to those known from the experimental data [8, 12, 14] at different pH and temperature values (see Table 2). The dissociation constants of phosphorylated dimer were far from experimentally obtained [8] (see Table 2). Possibly this is due to some conditions not taken into account or presence of some forms with bound substrate (PEP or Pyr), which can shift equilibrium in experiment. We have also compared the fitted rate constants of the dimerization and dissociation with the experimental estimates from [25] (Table 2). The value of the equilibrium constant of PEP dissociation from the monomer has the same order of magnitude as the experimentally [13] obtained value of the PEP dissociation constant of the C-terminal domain of the monomer (Table 2). Finally, *K*_*m*_ values for EI substrate PEP has been calculated and found to agree with published previously [5]. It can be seen from Table 2 that model error is not very sensitive to some of the constants: twice increase of *f* cannot be achieved, for example, even at very low values of some parameters. Nevertheless values of these parameters are given corresponding to the minimum of *f*.

Comparison of the values obtained for the constants of elementary processes (see Table S.1 of Supplement 5) enables us to draw several conclusions about the interaction of the enzyme with its substrates:

1. Phosphorylation leads to the stabilization of a dimer (*K*_45_^dEIP2^ < *K*_48_^dEIP^ ≪ *K*_1_^dEI^ ) at pH 7.0, *t* = 27°C.
2. There exists a positive cooperativity in binding of PEP to Nonphosphorylated and mono-phosphorylated dimer (*K*_12_ > *K*_14_, *K*_30_ > *K*_35_, *K*_26_ > *K*_31_).
3. Pyr binds with positive cooperativity to a phosphorylated dimer (*R*_18_ < *R*_22_, *R*_21_ < *R*_25_, *R*_24_ < *R*_28_) and with negative cooperativity to nonphosphorylated dimer (*R*_9_ < *R*_6_).
4. PEP and Pyr bind with negative cooperativity to a dimer (*R*_13_ > *R*_9_) and with positive cooperativity to a phosphorylated dimer (*R*_23_ < *R*_22_, *R*_37_ < *R*_28_).
5. For phosphorylation there exists a negative cooperativity (*K*_*p*_^*t*0^ > *K*_*q*_^*tp*^ at *p* = *q*).
6. The presence of PEP or Pyr on either of the subunits improves phosphorylation of the other subunit (*K*_*p*_^*t*0^ ≥ *K*_16_^*t*0^, *K*_*q*_^*tp*^ ≥ *K*_27_^*tp*^ at any *p* and *q*) but PEP decreases the rate of phosphorylation (*k*_17_ < *k*_16_, *k*_36_ < *k*_27_).
7. As has been shown in experiments [8, 10], PEP shifts equilibrium towards the formation of dimers: *K*_5_^dEI^ = 9.7 · 10^−8^ mM, *K*_3_^dEI^ = 8 · 10^−5^ mM, *K*_1_^dEI^ = 3.8 · 10^−4^ mM.
8. Singly phosphorylated dimer transfers the phosphate group to HPr at the highest rate and equilibrium constant (see constants *kh*_10_ and  *Kh*_*d*1_), but, when PEP is bound to a Nonphosphorylated subunit, HPr phosphorylation is not observed (*kh*_12_ = *kh*_14_ = *kh*_17_ = 0).
9. The model is sensitive to alteration of the parameters which are involved in dimerization; it proves the important regulatory role of this process in the cell.

#### 3.4.3. Dependence of the Phosphate Transfer Rate on Physiological PEP Concentrations

**Figure 9:**
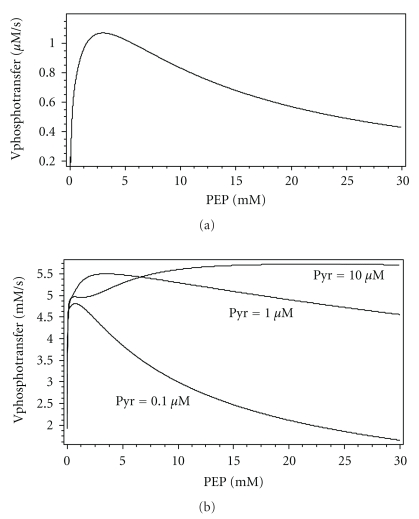
Dependence of the steady state HPr phosphorylation rate on the PEP concentration as calculated by the Model II. (a) in the absence of Pyr, (b) in presence of Pyr. Pyr concentrations are shown near the lines, concentrations of the enzymes are as given in the legend of Figure 7.

An unusual dependence of the rates of transfer of the phosphate group from EI to HPr on the PEP concentration also turned out to be the interesting result in studying the properties of the enzymatic model developed (Figure 9). At the values of the kinetic parameters found by fitting, this dependence has nonmonotonic character (the values of the EI and HPr concentrations used for calculations correspond approximately to the physiological ones [25]. Initially, the rate increases when increasing the concentration of PEP up to 5 mM; however, beyond this concentration, it begins to decrease (Figure 9(a)). Probably this is due to the influence of the binding of PEP to the Nonphosphorylated subunit on the rate of phosphorylation performed by the other subunit. As can be seen from the rate constants found (see Supplement 5 and conclusion (8) in Section 3.4.2) the presence of PEP on one of the subunits inhibits the phosphate transfer to HPr (i.e., inhibition by the substrate is observed). In this case, the presence of Pyr results in the abolition of this effect (see Figure 9(b)). Pyr molecules are likely to bind competitively to EI, not allowing PEP to bind to it and inhibit the transfer. Figure 5(a) presents the dependence of the rate of HPr phosphorylation on the concentrations of PEP and Pyr. It is seen that the phosphorylation rate reaches maximum values if both PEP and Pyr are present. If the ratio of Pyr to PEP is too high or too low, no HPr phosphorylation predicted by the model. This ratio reflects the energy state of the cell; hence, it should determine the rate of transport of the required PTS substrates into the cell.

#### 3.4.4. The Dependence of the Phosphate Transfer Rate on Low PEP Concentrations

**Figure 10:**
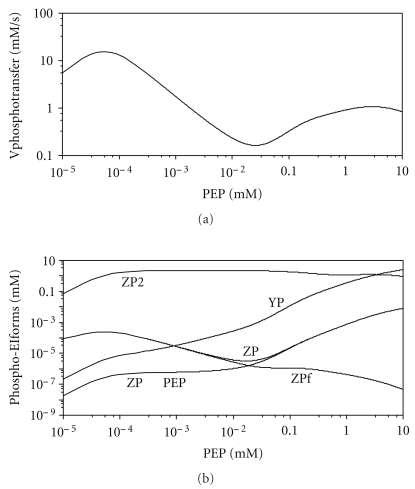
Behaviour of the system at low concentrations of PEP as calculated by the Model II. (a) dependence of the rate of HPr phosphorylation on the PEP concentration in the absence of Pyr; (b) dependence of the stationary concentrations of the different phosphorylated EI forms on PEP concentration: *ZP*∘PEP: singly phosphorylated protein with bound PEP; ZPf: singly phosphorylated protein without PEP. Other designations are as shown on Figure 4. At the PEP concentration equal zero, all concentrations of the phosphorylated EI-forms are equal zero (not shown).

Nonmonotonous behaviour of the enzyme at low PEP concentrations turned out to be another interesting property. At approximately 50 *μ*M PEP the flux of phosphate group to HPr was very low (0.2 *μ*M/s, Figure 10(a)). However, with decreasing PEP concentrations down to 0.1 *μ*M, the flux increased linearly to 10 *μ*M/s (Figure 10(a)). Unfortunately, no experimental data are available at such low concentrations of PEP, so it is difficult to judge the validity of this prediction.

 The reason for the nonmonotonous dependence of the phosphorylation rate on PEP is to be found in the complicated regulatory interactions that were discussed earlier. As has been stated, the main contribution to phosphate transfer is made by the singly phosphorylated dimer. To understand the reason why the values of the rates of phosphate transfer change, one should follow how the distribution between different enzymatic forms alters with changing PEP concentrations (see Figure 10(b)). Of course, in the absence of PEP, the flux of phosphate to HPr is zero. When PEP concentration increases to the submicromolar range, singly phosphorylated dimer appears, which results in an increase of the phosphate flux to the enzyme HPr. With a further increase of the PEP concentration, monomeric EI becomes phosphorylated and the *Z*P concentration decreases accordingly. However, with a further increase in PEP concentration, enzymatic forms with bound PEP appear. Since PEP has a negative effect on the rate of the second phosphorylation (conclusion (6) in Section 3.4.2), the greater part of EI remains in the *Z*P form. At the same time, a portion of *Z*P with bound PEP also increases, and the rate of phosphate transfer to HPr with these forms is equal to zero (conclusion (8)). Though the concentration of free *Z*P form does not decrease beyond 5 mM PEP, the rate of phosphate transfer starts to decrease. It was mentioned above, in Section 3.4.1, that PEP and Pyr concentrations can reach very low values. It can also be seen, from the analysis of the kinetic curves on the Figure 3 of [26] that during the first five seconds, while concentration of EI substrate PEP falls from 1.2 mM to 0.1 mM, the rate of glucose consumption remains unchanged and even slightly increases. This means that activity of PTS enzymes also increases with decreasing PEP. The results of our modelling can account for this effect by the complicated regulatory profile of the enzyme. 

It should be noted that considering the enzymatic properties, the use of our model at very low concentrations is possibly not sound: the rate equations were derived assuming that the binding of PEP to Enzyme I was much faster than any other reaction. In the case of low PEP concentrations, substrate limitation is possible and then our assumption could be wrong and could lead to a false description of the kinetics. Nevertheless, we choose to present this result, realizing that it needs experimental confirmation.

#### 3.4.5. Physiological Role of the Doubly Phosphorylated Dimer

**Figure 11:**
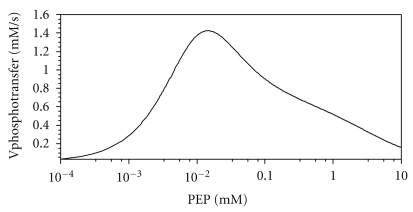
Dependence of the steady state HPr phosphorylation rate on the PEP concentration without the second dimer phosphorylation as calculated by the Model II. Concentrations of the enzymes are as given in the legend of Figure 7, Pyr = 0.

**Figure 12:**
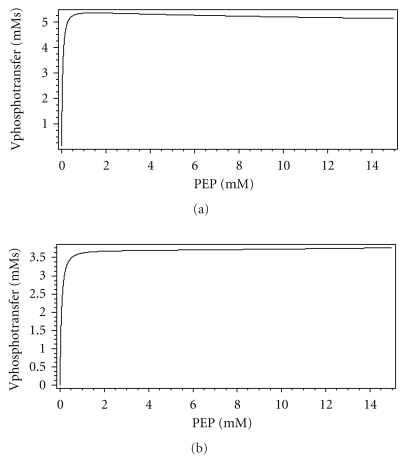
Dependence of the steady state HPr phosphorylation rate on the PEP concentration. (a) with and (b) without the second dimer phosphorylation in the presence of Pyr as calculated by the Model II. Concentrations of the enzymes are as given in the legend of Figure 7, Pyr = 0.2 mM.

After analysis of the systems behaviour, the physiological role of the *Z*P2 remained unclear to us. Calculations which include preventing second phosphorylation of the dimmer (*k*_20_ = *k*_27_ = *k*_36_ = 0) have shown that it does not affect the quantity of unphosphorylated monomers (*Y*), so it has not taken part in chemotaxis regulation. Studies of the phosphotransfer rate dependence on PEP without *Z*P2 at the obtained parameter values have shown that the rate of phosphotransfer would have been higher in this case and the curve would have had a different shape (Figure 11). It can be seen that in this case, the maximum of the HPr phosphorylation rate is in the range of 0.01–0.1 mM of PEP, while the minimum of this rate was in the presence of the second phosphorylation (Figure 10(a)). This may lead to the conclusion that existence of this dimer state may have some significance for regulation of the phosphotransfer rate in this range of PEP concentrations. Dependence of the stationary rate of phosphotransfer to HPr in presence of Pyr was also calculated. It can be seen from Figure 12 that in the model in which second phosphorylation is prevented, the rate of phosphotransfer increases at large PEP concentrations (Figure 12(b)), while in the model with *Z*P2 it decreases (Figure 12(a)). This difference may provide an explanation of the physiological role of *Z*P2. At high PEP concentrations, there may be no need for a fast phosphotransfer in the cell. Part of the enzyme is kept in this form, which is not able to transfer phosphate group to HPr rapidly. So, existence of the different EI-forms, which have different rates of phosphotransfer to HPr, may serve for the regulation of the phosphotransfer rate in different physiological conditions.

## 4. Conclusion

Modelling of the EI catalytic cycle with incorporation of most existing experimental data has been performed. With parameter values obtained by fitting, the major system behaviours have been investigated. The analysis of kinetic properties of EI at the parameters values identified in this study lead to some conclusions: (i) singly phosphorylated dimer promotes a high-speed transfer of phosphate to HPr, while a transfer of phosphate by a monomer is of importance in regulation of the chemotaxis; (ii) cooperativity is shown to exist with respect to the binding of substrates and products and to phosphorylation; (iii) the doubly phosphorylated dimer may have significance for phosphotransfer rate regulation. Model predicts also the nonmonotonic dependence of the rate of the phosphate transfer to HPr on the PEP concentration, which should be confirmed experimentally. 

The results obtained in this study are in line with modern ideas about the role of PTS in regulation of cellular metabolism. The various forms of EI are important for the regulation of different processes in the cell. Being sensitive to the substrate PEP and the product Pyr, Enzyme I, depending on the proportion of their concentrations, provides the needed flux of PTS sugars into the cell. On the other hand, the ratio of Pyr to PEP represents the energy status of the cell. These two metabolites constitute a link between glycolysis and the Krebs cycle and their ratio also determines how much phosphorylated EIIA, which is also involved in metabolism regulation (8), is present in the cell [4]. In addition, PEP is able to lose a phosphate group directly to ADP with the formation of ATP. So, in order to understand why the cell may need the presence of different mechanisms of phosphate transfer, analysis of the central metabolism as a whole using a kinetic model is required. This gives an insight into the role of EI in the cell.

## Supplementary Material

Supplementary material 1 gives description of the reduction of the model of elementary processes to differential equations for new variables and derivation of rate equations.
Supplementary material 2 contains expressions which have been used to decrease number of parameters: ratios of detailed balance, assumptions about equal parameter values.
In the Supplementary material 3 expressions for pH-dependence for different parameters are given.
Supplementary material 4 consists of figures which illustrate the goodness of fit and which were not added to the main text.
Supplementary material 5 contains table with values for both fitted parameter and parameters calculated through them using ratios of detailed balance.

## Figures and Tables

**Table 1:** Comparison of goodness of fit for different models with the help of AIC.

Type of model	*q*	*f*	AIC
(i) phosphotransfer by the monomer	82	1.13 · 10^4^	194

(ii) phosphotransfer by the doubly phosphorylated dimer	86	1.08 · 10^4^	202

(iii) phosphotransfer both by doubly and by singly phosphorylated dimer	87	9446	185

(iv) phosphotransfer by all forms of EI	90	516	179

**Table 2:** Comparison of the values of the parameters obtained by fitting and estimated from the experiments.

*T*	pH	Parameter	Experimentally obtained value	Value obtained by fitting	Range of sensitivity	Units	Ref.
25°C	6.5	*K*_1_^dEI^	210	170	5 · 10^−14^ ⋯ 2.4 · 10^4^	nM	
	7.5	*K*_1_^dEI^	1600	800	5 · 10^−14^ ⋯ 1.1 · 10^5^	nM	[14]
35°C	6.5	*K*_1_^dEI^	250	200	5 · 10^−14^ ⋯ 2.7 · 10^4^	nM
	7.5	*K*_1_^dEI^	3	0.9	5 · 10^−14^ ⋯ 130	*μ*M

37°C	7.1	*K*_1_^dEI^	0.51	0.5	5 · 10^−14^ ⋯ 75	*μ*M	[12]

23°C	6.5	*K*_1_^dEI^	0.23	0.16	5 · 10^−14^ ⋯ 23	*μ*M	
	7.5	*K*_1_^dEI^	0.32	0.77	5 · 10^−14^ ⋯ 100	*μ*M	[8]
	6.5	*K*_45_^dEIP2^	1.2	5 · 10^−4^	2 · 10^−4^ ⋯ 1.4 · 10^−2^	*μ*M
	7.5	*K*_45_^dEIP2^	3.7	2.3 · 10^−3^	8 · 10^−4^ ⋯ 6.6 · 10^−2^	*μ*M

37°C	6.5	* 1/k_1_*	0.004	0.003	9 · 10^−4^ ⋯ 7 · 10^−3^	s^−1^	[25]
		* k_45_*	0.01	0.43	0 ⋯ 1.1	s^−1^	

25°C	7.5	*K*_2_	0.2	0.37	2 · 10^−8^ ⋯ 1000	mM	[13]
25°C	7.2	*K*_*m*_^PEP^	0.18 mM	0.25 mM	—	—	[5]
