# Supplementary material for: EI of the Phosphotransferase System of Escherichia coli: Mathematical Modeling Approach to Analysis of Its Kinetic Properties
Source: J Biophys. 2011 Mar 20;2011:579402. doi: 10.1155/2011/579402 (PMC3116621; doi:10.1155/2011/579402)
Supplement: Supplementary file 1 — Supplementary material 1 gives description of the reduction of the model of elementary processes to differential equations for new variables and derivation of rate equations. Supplementary material 2 contains expressions which have been used to decrease number of parameters: ratios of detailed balance, assumptions about equal parameter values. In the Supplementary material 3 expressions for pH-dependence for different parameters are given. Supplementary material 4 consists of figures which illustrate the goodness of fit and which were not added to the main text. Supplementary material 5 contains table with values for both fitted parameter and parameters calculated through them using ratios of detailed balance. [file 579402.f1.pdf]

Supplementary material 1

A system of equations corresponds to the schemes in Figs. 1-3 in the main text and contains 26 differential equations for the changing of the concentration in every EI state, one law of conservation of total EI concentration, differential equations for PEP, Pyr, P_i and HPr, one law of conservation of total amount of HPr, laws of conservation of phosphate and "carbonic base". The right-hand member of the equation indicates a sum of the rates of all elementary processes in which this form is involved. The expressions for these rates were written according to the mass action law (see below):

$$\left.\begin{aligned}
&v_i = k_i(EI^2 - D \cdot K_i^{dEI}), && \text{i=1,3,5,7,8,11}\\
&v_k = k_k(DP - \frac{EI \cdot EIP}{K_k^{dEIP}}), && \text{k=47-55}\\
&v_s = k_s(DP_2 - \frac{EIP \cdot EIP}{K_s^{dEIP2}}), && \text{s=39, 40, 41, 42,44,45}\\
&v_p = k_p(DPEP - \frac{DPPyr}{K_p^{t0}}), && \text{p=15-17}\\
&v_q = k_q(DPPEP - \frac{DP_2Pyr}{K_q^{tp}}), && \text{q=20,27,36}\\
&v_r = k_r(EPEP - \frac{E \cdot PEP}{K_r}), && \text{r =2, 10,12,14, 26,29,30,31,33, 35,38, 43}\\
&v_u = k_u(EPyr - \frac{E \cdot Pyr}{R_u}), && \text{u=4,6,9,13,18, 21-25, 28, 34,37, 46}\\
&vh_l = kh_l \cdot (EIP \cdot HPr - \frac{EI \cdot HPrP}{Kh_l}), && \text{l=1-3}\\
&vh_m = kh_m \cdot (DP \cdot HPr - \frac{D \cdot HPrP}{Kh_m^{d1}}), && \text{m=10-18}\\
&vh_n = kh_n \cdot (DP_2 \cdot HPr - \frac{DP_2 \cdot HPrP}{Kh_n^{d}}), && \text{n=4-9,}
\end{aligned}\right\} (S.1)$$

Here EI is any form of a non-phosphorylated monomer. EIP is any form of a phosphorylated monomer, D is any form of a non-phosphorylated dimer, DP is any form of a phosphorylated dimer, DP_2 is any form of a doubly-phosphorylated dimer, EPEP is any form of the enzyme with bound PEP, EPyr is any form of the enzyme with bound Pyr; the constants of dimer dissociation: K_i^{dEI} correspond to non-phosphorylated dimer, K_k^{dEIP} correspond to phosphorylated dimer, K_s^{dEIP2} correspond to doubly-phosphorylated dimer; the equilibrium

constants of dimer phosphorylation: K_p^{t0} correspond to phosphorylation of the non-phosphorylated dimer, K_q^{tp} correspond to phosphorylation of the singly-phosphorylated dimer; the constants of substrates dissociation: K_r correspond to dissociation of the PEP, R_u is the same for Pyr (symbol R for Pyr dissociation constant is used for to make clear conclusions in the paragraph "Studying the obtained values of the parameters"); the equilibrium constants of the HPr phosphorylation: Kh_l correspond to phosphorylation by a monomer, Kh_m^{d1} correspond to phosphorylation by a phosphorylated dimer, Kh_n^{d} correspond to phosphorylation by a doubly-phosphorylated dimer. Symbols and indices of the constants of rates correspond to those given in Figs. 1-3.

The example of rate notation is given below.

In order to simplify and reduce the amount of equations we divided the states of EI into 5 groups by features of phosphate group presence and the state of oligomerization: a non-phosphorylated monomer, a non-phosphorylated dimer, a singly-phosphorylated dimer, a doubly-phosphorylated dimer, a phosphorylated monomer. Thus, the state of the enzyme inside any group changes due to the binding of PEP or Pyr, the transfer from one group to another is carried out through reactions of dimerization (dissociation) and reactions of phosphorylation

In conformity with assumption (3) in the section "Known experimental data and hypotheses used for model development" in the main text, reactions of substrates binding (PEP, Pyr) run much faster than the transfer of phosphate group to EI or dimerization [14]. Each of these reactions can be considered to be in quasi-equilibrium.

So, it is possible to turn to new variables – total concentrations of the enzyme's states belonging to one of the groups. Here, the rates of the changing of these concentrations, as already mentioned above, are determined by dimerization (dissociation) and phosphorylation reaction rates.

In order to demonstrate the transformation from a complete to a reduced catalytic cycle in detail, let us consider one section in the reaction scheme, where a non-phosphorylated monomer (EI^f) and a complex of this monomer with PEP (EI^{PEP}) and Pyr (EI^{Pyr}) are involved (Fig. 1)

We can demonstrate with this example how we proceeded to new variables as well as how previous variables (concentrations of different forms of the enzyme) were expressed through concentrations of new variables.

Here, we write equations that describe changes of former variables in time. The concentrations of different forms of a non-phosphorylated monomer alter because of the processes of substrate binding, the formation and decay of non-phosphorylated and once phosphorylated dimer and HPr phosphorylation (see Fig. 1 in the main text and Fig. 1 in Appendix).

$$\left.\begin{aligned} \frac{dEI^f}{dt} &= -2v_1 - v_3 - v_7 - v_2 - v_4 + vh_2 + v_{47} + v_{48} + v_{49} \\ \frac{dEI^{PEP}}{dt} &= -v_3 - 2v_5 - v_{11} + v_2 + vh_3 + v_{50} + v_{51} + v_{52} \\ \frac{dEI^{Pyr}}{dt} &= -v_7 - 2v_8 - v_{11} + v_4 + vh_1 + v_{53} + v_{54} + v_{55} \end{aligned}\right\} (S.2)$$

The numbers of reactions correspond to the numbers of reactions in the Figures. In accordance with the assumptions, made above, the reactions 2 and 4 are rapid and others are slow.

Writing the sum of all three equations instead of the first equation gives a new system:

$$\left.\begin{aligned} \frac{d(EI^f + EI^{PEP} + EI^{Pyr})}{dt} &= -2V_1 + V_5 + V_6 \\ \frac{dEI^{PEP}}{dt} &= -v_3 - 2v_5 - v_{11} + v_2 + vh_3 + v_{50} + v_{51} + v_{52} \\ \frac{dEI^{Pyr}}{dt} &= -v_7 - 2v_8 - v_{11} + v_4 + vh_1 + v_{53} + v_{54} + v_{55} \end{aligned}\right\} (S.3)$$

where,

$$\left.\begin{aligned}
V_1 &= \sum_{i=1,3,5,7,8,11} v_i \\
V_5 &= \sum_{i=1}^{3} vh_i \\
V_6 &= \sum_{i=47}^{55} v_i
\end{aligned}\right\} \quad (S.4)$$

Consider the second equation in S.3

$$\frac{dEI^{PEP}}{dt} = -v_3 - 2v_5 - v_{11} + v_2 + vh_3 + v_{50} + v_{51} + v_{52}$$

Since the rate v_2 in the second equation can be expressed as:

$$v_2 = k_2 \cdot (EI^f \cdot PEP - EI^{PEP} \cdot K_2) \quad (S.5)$$

the second equation can be rewritten as follows:

$$\frac{dEI^{PEP}}{dt} = -v_3 - 2v_5 - v_{11} + k_2 \cdot (EI^f \cdot PEP - EI^{PEP} \cdot K_2) + vh_3 + v_{50} + v_{51} + v_{52} \quad (S.6)$$

Dividing the left and the right side of the equation by k_2 gives:

$$\frac{1}{k_2} \cdot \frac{dEI^{PEP}}{dt} = \frac{vh_3 + v_{50} + v_{51} + v_{52} - v_3 - 2v_5 - v_{11}}{k_2} + (EI^f \cdot PEP - EI^{PEP} \cdot K_2) \quad (S.7)$$

Proceed to the limit at $k_2 \to \infty$:

$$\left.\begin{aligned}
&\frac{1}{k_2} \cdot \frac{dEI^{PEP}}{dt} \to 0 \\
&\frac{vh_3 + v_{50} + v_{51} + v_{52} - v_3 - 2v_5 - v_{11}}{k_2} \to 0
\end{aligned}\right. \quad (S.8)$$

Therefore,

$$\begin{aligned}
&EI^f \cdot PEP - EI^{PEP} \cdot K_2 \to 0, \\
&EI^{PEP} = \frac{EI^f \cdot PEP}{K_2}
\end{aligned} \quad (S.9)$$

It is analogous for the forms of the enzyme with the bound Pyr from the third equation in (S.3) at $v_4 \to \infty$

$$EI^{Pyr} = \frac{EI^f \cdot Pyr}{R_4} \quad (S.10)$$

Pass to new variables:

$$Y = EI^f + EI^{PEP} + EI^{Pyr} \qquad \text{(S.11)}$$

Taking into account the formulae (S.9-11), the former variables are expressed through *Y* as follows:

$$\left.\begin{aligned} EI^f &= \frac{Y}{\Delta_Y} \\ EI^{PEP} &= \frac{Y}{\Delta_Y}\cdot\frac{PEP}{K_2} \\ EI^{Pyr} &= \frac{Y}{\Delta_Y}\cdot\frac{Pyr}{R_4} \end{aligned}\right\} \qquad \text{(S.12)}$$

where,

$$\Delta_Y = 1 + \frac{PEP}{K_2} + \frac{Pyr}{R_4}$$

So, we moved from a system of three differential equations to a system of two algebraic equations and one differential one for the variable Y, the value of which changes only due to dimerization of EI (dissociation of the dimer) and HPr phosphorylation:

$$\left.\begin{aligned} \frac{dY}{dt} &= -2V_1 + V_5 + V_6 \\ EI^{PEP} &= \frac{Y\cdot PEP}{\Delta_Y\cdot K_2} \\ EI^{Pyr} &= \frac{Y\cdot Pyr}{\Delta_Y\cdot R_4} \end{aligned}\right\} \qquad \text{(S.13)}$$

Now expressions for the rates V_1, V_5 and V_6 can be rewritten in accordance with the formulae (S.1) and (S.4). We will do it for V_1 as an example. So:

Consider the rate V_1, in compliance with formulae (S.1):

$$\begin{aligned} V_1 = {} & k_1((EI^f)^2 - D^f\cdot K_1^{dEI}) + k_3(EI^f\cdot EI^{PEP} - D^{PEP}\cdot K_3^{dEI}) + k_5((EI^{PEP})^2 - D^{PEP2}\cdot K_5^{dEI}) + \\ & + k_7(EI\cdot EI^{Pyr} - D^{Pyr}\cdot K_7^{dEI}) + k_8((EI^{Pyr})^2 - D^{Pyr2}\cdot K_8^{dEI}) + k_{11}(EI^{PEP}\cdot EI^{Pyr} - D^{PEPPyr}\cdot K_{11}^{dEI}) \end{aligned} \qquad \text{(S.14)}$$

Here, D^{index} is the non-phosphorylated dimmer concentration, the upper indices denote bound ligands, so that D^f is the free dimer concentration; a dimer with bound substrates: D^{PEP} - with PEP, D^{Pyr} - with Pyr, D^{PEP2} - with two molecules of PEP, D^{Pyr2} - with two molecules of

Pyr, D^{PEPPyr} - with PEP and Pyr. In the formula (S.14) the former variables are used, so we ought to turn to new variables. EI^{PEP} and EI^{Pyr} are expressed in accordance with the formulae (S.13). In a similar manner it is possible to show that the forms of the dimer can be expressed as:

$$\left.\begin{aligned} D^{PEP} &= \frac{Z \cdot PEP}{\Delta_z \cdot K_{12}} \\ D^{Pyr} &= \frac{Z \cdot Pyr}{\Delta_z \cdot R_9} \\ D^{PEP2} &= \frac{Z \cdot PEP^2}{\Delta_z \cdot K_{12} \cdot K_{14}} \\ D^{Pyr2} &= \frac{Z \cdot Pyr^2}{\Delta_z \cdot R_9 \cdot R_6} \\ D^{PEPPyr} &= \frac{Z \cdot PEP \cdot Pyr}{\Delta_z \cdot K_{12} \cdot R_{13}} \end{aligned}\right\} \quad (S.15)$$

where,

$$\Delta_Z = 1 + \frac{PEP}{K_{12}} \cdot (1 + \frac{PEP}{K_{14}} + \frac{Pyr}{R_{13}}) + \frac{Pyr}{R_9} \cdot (1 + \frac{Pyr}{R_6})$$

The reaction rate V_1 then can be written as:

$$V_1 = r_1 \cdot Y^2 - r_{-1} \cdot Z \,, \quad (S.16)$$

where,

$$r_1 = (k_1 + k_3 \cdot \frac{PEP}{K_2} + k_7 \cdot \frac{Pyr}{R_4} + k_5 \cdot \frac{(PEP)^2}{(K_2)^2} + k_8 \cdot \frac{(Pyr)^2}{(R_4)^2} + k_{11} \cdot \frac{PEP}{K_2} \cdot \frac{Pyr}{R_4}) / \Delta_Y^2 \quad (S.17)$$

$$r_{-1} = (k_1 + k_3 \cdot K_3^{dEI} \cdot \frac{PEP}{K_{12}} + k_7 \cdot K_7^{dEI} \cdot \frac{Pyr}{R_9} + k_5 \cdot K_5^{dEI} \cdot \frac{(PEP)^2}{K_{12} \cdot K_{14}} + k_8 \cdot K_8^{dEI} \cdot \frac{(Pyr)^2}{R_6 \cdot R_9} + k_{11} \cdot K_{11}^{dEI} \cdot \frac{PEP}{K_{12}} \cdot \frac{Pyr}{R_{13}}) / \Delta_Z \quad (S.18)$$

However, according to the relations of detailed balance

$$\frac{K_3^{dEI}}{K_{12}} = \frac{K_1^{dEI}}{K_2} \,,$$

$$\frac{K_7^{dEI}}{R_6} = \frac{K_1^{dEI}}{R_4} \quad etc.$$

Substituting these expressions in the formula (S.14) it can be written in the more simple form -

$$r_{-1} = r_1 \cdot \Delta_Y^2 \cdot K_1^{dEI} / \Delta_Z \quad (S.18')$$

The rates V_5 and V_6 can be described in the same fashion.

Other parts of the scheme are described similarly. Since at any stage the binding of PEP and Pyr proceed much faster, then it is reasonable to use differential equations when describing variables, the value of which change in the course of the following processes: the formation and decay of EI dimer (V_1, V_6, V_4), the first and the second phosphorylation of EI dimer (V_2, V_3), phosphorylation of HPr with a monomer (V_5) and dimer (V_7, V_8). Thus, all the states of EI enzyme are to be divided into the following groups: a monomer, a phosphorylated monomer, a dimer, a singly-phosphorylated dimer, a doubly-phosphorylated dimer. In this case, analogous to the example considered above (formulae S.4), the rates of changing of these values are equal to the sum of the rates of elementary reactions.

$$\left.\begin{aligned} V_2 &= \sum_{i=15}^{17} v_i \\ V_3 &= \sum_{i=20,27,36} v_i \\ V_4 &= \sum_{\substack{i=39 \\ i\neq 43}}^{45} v_i \\ V_7 &= \sum_{i=4}^{9} vh_i \\ V_8 &= \sum_{i=10}^{18} vh_i \end{aligned}\right\} \qquad \text{(S.19)}$$

A new reduced catalytic cycle is given in Fig. 2.

Thus, the kinetic model describes the following variables:

a monomer Y with or without bound ligand (PEP or Pyr):

$$Y = EI^f + EI^{PEP} + EI^{Pyr}; \qquad \text{(S.20.a)}$$

a dimer Z with or without bound ligand (PEP and/or Pyr), in every possible arrangement :

$$Z = D^f + D^{PEP} + D^{Pyr} + D^{PEP2} + D^{Pyr2} + D^{PEPPyr}; \qquad \text{(S.20.b)}$$

Phosphorylated dimers with or without bound ligand (PEP and/or Pyr), upper indices denote ligands at subunits: the first index denotes ligand at the phosphorylated subunit, the second one denotes ligand at the other subunit:

$$ZP = DP^{f} + DP^{PEP0} + DP^{Pyr0} + DP^{0PEP} + DP^{0Pyr} + DP^{PEP2} + DP^{Pyr2} + DP^{PEPPyr} + DP^{PyrPEP};$$

(S.20.c)

a doubly-phosphorylated dimer with or without bound ligand (PEP and/or Pyr):

$$ZP_2 = DP_2^{\,f} + DP_2^{\,PEP} + DP_2^{\,Pyr} + DP_2^{\,PEP2} + DP_2^{\,Pyr2} + DP_2^{\,PEPPyr}; \quad \text{(S.20.d)}$$

A phosphorylated monomer with or without bound ligand (PEP or Pyr):

$$YP = EIP^{f} + EIP^{PEP} + EIP^{Pyr}; \quad \text{(S.20.e)}$$

Taking into account the quasi-equilibrium approximation these relations can be rewritten through concentrations of free enzyme forms and substrates:

$$\left.\begin{array}{l} Y = EI \cdot \Delta_Y \\ Z = D \cdot \Delta_Z \\ ZP = DP \cdot \Delta_{ZP} \\ ZP_2 = DP_2 \cdot \Delta_{ZP_2} \\ YP = EIP \cdot \Delta_{YP} \end{array}\right\} \quad \text{(S.21)}$$

where

$$\Delta_Y = 1 + \frac{PEP}{K^2} + \frac{Pyr}{R^4}$$

$$\Delta_Z = 1 + \frac{PEP}{K_{12}} \cdot \left(1 + \frac{PEP}{K_{14}} + \frac{Pyr}{R_{13}}\right) + \frac{Pyr}{R_9} \cdot \left(1 + \frac{Pyr}{R_6}\right)$$

$$\Delta_{ZP} = 1 + \frac{Pyr}{R_{25}} + \frac{PEP}{K_{26}} + \frac{Pyr}{R_{22}} \cdot \left(1 + \frac{PEP}{K_{19}} + \frac{Pyr}{R_{18}}\right) + \frac{PEP}{K_{30}} \cdot \left(1 + \frac{PEP}{K_{35}} + \frac{Pyr}{R_{34}}\right) \quad \text{(S.22)}$$

$$\Delta_{ZP2} = 1 + \frac{PEP}{K_{33}} \cdot \left(1 + \frac{PEP}{K_{38}}\right) + \frac{Pyr}{R_{28}} \cdot \left(1 + \frac{Pyr}{R_{24}} + \frac{PEP}{K_{32}}\right)$$

$$\Delta_{YP} = 1 + \frac{PEP}{K_{43}} + \frac{Pyr}{R_{46}}$$

Different forms of HPr:

HPr is a free enzyme

HPrP is a phosphorylated enzyme

Moreover, the model takes into account laws of conservation for the amount of EI and HPr.

$$Y + 2 \cdot Z + 2 \cdot ZP + 2 \cdot ZP2 + YP = EI_{total}$$
$$HPrP + HPr = HPr_{total} \tag{S.23}$$

Here, EI_{total} and HPr_{total} are pools of EI monomers and HPr, respectively.

The rates of reactions in this model are calculated analogous to the example given above (formulae (S.14-18′), so we omit the derivation:

The rate of the first phosphorylation

$V_2 = r_2 \cdot Z - r_{-2} \cdot ZP$, where

$$r_2 = \frac{PEP}{K_{12}} \cdot (k_{17} \cdot \frac{PEP}{K_{14}} + k_{16} + k_{15} \cdot \frac{Pyr}{R_{13}}) / \Delta_Z \tag{S.24.1}$$

$$r_{-2} = r_2 \cdot \Delta_Z \cdot Pyr \cdot K_{12} / (PEP \cdot R_{22} \cdot K_{16}^{t0} \cdot \Delta_{ZP})$$

The rate of the second phosphorylation

$V_3 = r_3 \cdot ZP - r_{-3} \cdot ZP2$, where

$$r_3 = \frac{PEP}{K_{26}} \cdot (k_{36} \cdot \frac{PEP}{K_{35}} + k_{27} + k_{20} \cdot \frac{Pyr}{R_{23}}) / \Delta_{ZP} \tag{S.24.2}$$

$$r_{-3} = r_3 \cdot \Delta_{ZP} \cdot Pyr \cdot K_{30} / (PEP \cdot R_{28} \cdot K_{27}^{tp} \cdot \Delta_{ZP2})$$

The rate of dissociation of the singly-phosphorylated dimer

$V_6 = r_6 \cdot ZP - r_{-6} \cdot YP \cdot Y$, where

$$\mathrm{r}_6 = \left[k_{48} + k_{54} \cdot \frac{Pyr}{R_{25}} + k_{51} \cdot \frac{PEP}{K_{26}} + \frac{Pyr}{R_{22}} \cdot (k_{47} + k_{50} \cdot \frac{PEP}{K_{19}} + k_{53} \cdot \frac{Pyr}{R_{18}}) + \frac{PEP}{K_{30}} \cdot (k_{49} + k_{52} \cdot \frac{PEP}{K_{35}} + k_{55} \cdot \frac{Pyr}{R_{34}}) \right] / \Delta_{ZP}$$
$$r_{-6} = r_6 \cdot \Delta_{ZP} / (\Delta_Y \cdot \Delta_{YP} \cdot K_{48}^{dEIP}) \tag{S.24.3}$$

The rate of dissociation of the doubly-phosphorylated dimer

$V_4 = r_4 \cdot ZP2 - r_{-4} \cdot YP^2$, where

$$r_4 = \left[k_{45} + \frac{PEP}{K_{33}} \cdot (k_{40} + k_{39} \cdot \frac{PEP}{K_{38}}) + \frac{Pyr}{R_{28}} \cdot (k_{42} + k_{24} \cdot \frac{Pyr}{R_{24}} + k_{41} \cdot \frac{PEP}{K_{32}}) \right] / \Delta_{ZP2} \tag{S.24.4}$$

$$r_{-4} = r_4 \cdot \Delta_{ZP2} / ({\Delta_Y}^2 \cdot K_{45}^{dEIP2})$$

The rate of the transfer of phosphate by a monomer to HPr

$V_5 = r_5 \cdot HPr \cdot YP - r_{-5} \cdot HPrP \cdot Y$,   where

$$r_5 = (kh_3 \cdot \frac{PEP}{K_{43}} + kh_2 + kh_1 \cdot \frac{Pyr}{R_{46}}) / \Delta_{YP} \qquad \text{(S.24.5)}$$

$$r_{-5} = r_5 \cdot \Delta_{YP} / (\Delta_Z \cdot Kh_2)$$

The rate of the transfer of phosphate by a doubly-phosphorylated dimer to HPr

$$V_7 = r_7 \cdot HPr \cdot ZP2 - r_{-7} \cdot HPrP \cdot ZP$$

$$r_7 = \left[kh_4 + \frac{PEP}{K_{33}} \cdot (kh_5 + kh_6 \cdot \frac{PEP}{K_{38}}) + \frac{Pyr}{R_{28}} \cdot (kh_7 + kh_8 \cdot \frac{Pyr}{R_{24}} + kh_9 \cdot \frac{PEP}{K_{32}}) \right] / \Delta_{ZP2} \qquad \text{(S.24.6)}$$

$$r_{-7} = r_7 \cdot \Delta_{ZP2} / (\Delta_{ZP} \cdot Kh_4^d)$$

The rate of the transfer of phosphate by a singly-phosphorylated dimer to HPr

$$V_8 = r_8 \cdot HPr \cdot ZP - r_{-8} \cdot HPrP \cdot Z$$

$$r_8 = \left[kh_{10} + kh_{11} \cdot \frac{Pyr}{R_{25}} + kh_{12} \cdot \frac{PEP}{K_{26}} + \frac{Pyr}{R_{22}} \cdot (kh_{13} + kh_{14} \cdot \frac{PEP}{K_{19}} + kh_{15} \cdot \frac{Pyr}{R_{18}}) + \frac{PEP}{K_{30}} \cdot (kh_{16} + kh_{17} \cdot \frac{PEP}{K_{35}} + kh_{18} \cdot \frac{Pyr}{R_{34}}) \right] / \Delta_{ZP}$$

$$r_{-8} = r_8 \cdot \Delta_{ZP} / (\Delta_Z \cdot Kh_{10}^{d1}) \qquad \text{(S.24.7)}$$

HPrP hydrolysis

$$V_9 = r_9 \cdot HPrP - r_{-9} \cdot HPr \cdot P \qquad \text{(S.24.8)}$$

As in some experiments PEP concentration was comparable to the enzyme concentration, the change of this concentration cannot be neglected. In the initial system (1st paragraph), changes of PEP and Pyr concentrations were described by the differential equations, in the right part of which was the sum of the velocities of elementary processes. In order to describe the change of PEP and Pyr concentration without considering separate steps of binding, we wrote two laws of conservation:

$PEP + PTSP + EI \circ PEP + P_i = PEP(0) + PTSP(0)$ – is the law of conservation for a phosphate group

$Pyr = Pyr(0) + (PTSP - PTSP0) - EI \circ Pyr$ - is the law of conservation for carbonic bases quantity.

Here, PTSP is a total concentration of phosphorylated proteins of EI and HPr:

$PTSP = ZP + 2 \cdot ZP2 + YP + HPrP$;

$PEP(0)$, $Pyr(0)$, $PTSP(0)$ denote initial concentrations of PEP, Pyr, PTSP respectively, P_i is inorganic phosphate, $EI \circ PEP$ and $EI \circ Pyr$ are the total concentrations of the forms of the enzyme to which metabolites PEP and Pyr are bound, respectively. Here the concentration of the PEP bound to the enzyme is calculated as a sum of concentrations of all the states with bound PEP. These states are expressed through novel variables in accordance with the formulae from the system (S.13, 15):

$$EI \circ PEP = \frac{Y}{\Delta_Y} \cdot \frac{PEP}{K_2} + \frac{Z}{\Delta_Z} \cdot \frac{PEP}{K_{12}} \cdot (1 + 2 \cdot \frac{PEP}{K_{14}} + \frac{Pyr}{R_{13}}) + \frac{ZP}{\Delta_{ZP}} \cdot \left[\frac{PEP}{K_{26}} + \frac{Pyr}{R_{22}} \cdot \frac{PEP}{K_{19}} + \frac{PEP}{K_{30}} \cdot (1 + 2 \cdot \frac{PEP}{K_{35}} + \frac{Pyr}{R_{34}} [illegible] \right.$$
$$\left. + \frac{ZP2}{\Delta_{ZP2}} \cdot \left[\frac{PEP}{K_{33}} \cdot (1 + 2 \cdot \frac{PEP}{K_{38}}) + \frac{Pyr}{R_{28}} \cdot \frac{PEP}{K_{37}} \right] + \frac{YP}{\Delta_{YP}} \cdot \frac{PEP}{K_{43}} \right.$$

(S.25.1)

Similarly, Pyr bound to the enzyme is calculated:

$$EI \circ Pyr = \frac{Y}{\Delta_Y} \cdot \frac{Pyr}{R_4} + \frac{Z}{\Delta_Z} \cdot \left[\frac{PEP}{K_{12}} \cdot \frac{Pyr}{R_{13}} + \frac{Pyr}{R_9} \cdot (1 + 2 \cdot \frac{Pyr}{R_6}) \right] + \frac{ZP}{\Delta_{ZP}} \cdot \left[\frac{Pyr}{R_{25}} \cdot (1 + \frac{PEP}{K_{29}}) + \frac{Pyr}{R_{22}} \cdot (1 + \frac{PEP}{K_{19}} + 2 \cdot \frac{Py[illegible]}{R_{1}[illegible]} \right.$$
$$\left. + \frac{ZP2}{\Delta_{ZP2}} \cdot \frac{Pyr}{R_{28}} \cdot (1 + \frac{Pyr}{R_{24}} + \frac{PEP}{K_{37}}) + \frac{YP}{\Delta_{YP}} \cdot \frac{Pyr}{R_{46}} \right.$$

(S.25.2)

Thus, our model contains 6 differential equations and 4 laws of conservation.

$$\frac{dY}{dt} = -2 \cdot V_1 + V_5 + V_6$$
$$\frac{dZ}{dt} = V_1 - V_2 + V_8$$
$$\frac{dZP}{dt} = V_2 - V_3 - V_8 + V_7 - V_6 \qquad \text{(S.26)}$$
$$\frac{dYP}{dt} = 2 \cdot V_4 - V_5 + V_6$$
$$\frac{dHPrP}{dt} = V_5 + V_7 + V_8 - V_9$$
$$\frac{dP_i}{dt} = V_9$$

$$Y + 2 \cdot Z + 2 \cdot ZP + 2 \cdot ZP2 + YP = EI_{total}$$
$$PEP + PTSP + EI \circ PEP + P_i = PEP(0) + PTSP(0)$$
$$Pyr = Pyr(0) + (PTSP - PTSP0) - EI \circ Pyr$$
$$HPrP + HPr = HPr_{total}$$

Supplementary material 2.

In the list (Table S.1) the constants marked with asterisk can be expressed through independent constants (not marked with asterisk) with the use of ratios of detailed balance.

So,

$$K_{10} = R_{13} \cdot K_{12} / R_9$$
$$R_{18} = R_{13} \cdot K_{16}^{t0} / K_{15}^{t0}$$
$$K_{19} = K_{14} \cdot K_{15}^{t0} / K_{17}^{t0}$$
$$R_{21} = R_{18} \cdot R_{22} / R_{25}$$
$$R_{23} = R_{22} \cdot K_{19} / K_{26}$$
$$R_{24} = R_{23} \cdot K_{27}^{tp} / K_{20}^{tp}$$
$$K_{32} = K_{31} \cdot K_{27}^{tp} / K_{36}^{tp}$$
$$R_{34} = R_{25} \cdot K_{29} / K_{30}$$
$$K_{35} = K_{31} \cdot K_{26} / K_{30}$$
$$R_{37} = K_{32} \cdot R_{28} / K_{33}$$
$$K_3^{dEI} = K_1^{dEI} \cdot K_{12} / K_2$$
$$K_5^{dEI} = K_3^{dEI} \cdot K_{14} / K_2$$
$$K_7^{dEI} = K_1^{dEI} \cdot R_9 / R_4$$
$$K_8^{dEI} = K_7^{dEI} \cdot R_6 / R_4$$
$$K_{39}^{dEIP2} = K_{40}^{dEIP2} \cdot K_{38} / K_{43}$$
$$K_{40}^{dEIP2} = K_{45}^{dEIP2} \cdot K_{33} / K_{43}$$
$$K_{42}^{dEIP2} = K_{45}^{dEIP2} \cdot R_{28} / R_{46}$$
$$K_{44}^{dEIP2} = K_{42}^{dEIP2} \cdot R_{24} / R_{46}$$
$$K_{47}^{dEIP} = K_{48}^{dEIP} \cdot R_{22} / R_{46}$$
$$K_{49}^{dEIP} = K_{48}^{dEIP} \cdot K_{30} / K_{43}$$
$$K_{50}^{dEIP} = K_{47}^{dEIP} \cdot K_{19} / K_2$$
$$K_{51}^{dEIP} = K_{48}^{dEIP} \cdot K_{26} / K_2$$
$$K_{52}^{dEIP} = K_{49}^{dEIP} \cdot K_{35} / K_2$$
$$K_{53}^{dEIP} = K_{47}^{dEIP} \cdot R_{18} / R_{46}$$
$$K_{54}^{dEIP} = K_{48}^{dEIP} \cdot R_{25} / R_4$$
$$K_{55}^{dEIP} = K_{54}^{dEIP} \cdot K_{21} / K_{46}$$
$$Kh_1 = R_{46} \cdot Kh_2 / R_4$$
$$Kh_3 = K_{43} \cdot Kh_2 / K_2 \qquad \text{(S.27)}$$
$$Kh_5{}^d = K_{33} \cdot Kh_4^d / K_{26}$$

$$Kh_{5'}^{d} = K_{33} \cdot Kh_4^d / K_{30}$$
$$Kh_6^{d} = K_{38} \cdot Kh_5^{d} / K_{31}$$
$$Kh_7^{d} = R_{28} \cdot Kh_4^d / R_{25}$$
$$Kh_{7'}^{d} = R_{28} \cdot Kh_4^d / R_{22}$$
$$Kh_8^{d} = R_{24} \cdot Kh_7^{d} / R_{25}$$
$$Kh_9^{d} = R_{37} \cdot Kh_5^{d} / R_{23}$$
$$Kh_{9'}^{d} = K_{32} \cdot Kh_7^{d} / K_{29}$$
$$Kh_{11}^{d1} = R_{25} \cdot Kh_{10}^{d1} / R_9$$
$$Kh_{12}^{d1} = K_{26} \cdot Kh_{10}^{d1} / K_{12}$$
$$Kh_{13}^{d1} = R_{22} \cdot Kh_{10}^{d1} / R_9$$
$$Kh_{14}^{d1} = K_{23} \cdot Kh_{12}^{d1} / K_{13}$$
$$Kh_{15}^{d1} = R_{21} \cdot Kh_{11}^{d1} / R_6$$
$$Kh_{16}^{d1} = K_{30} \cdot Kh_{10}^{d1} / K_{12}$$
$$Kh_{17}^{d1} = K_{35} \cdot Kh_{16}^{d1} / K_{14}$$
$$Kh_{18}^{d1} = K_{29} \cdot Kh_{11}^{d1} / K_{13}$$

In accordance with the assumption (1) mentioned in the part «Known experimental data and hypotheses used for the model development» in the main text, the presence of Pyr at the subunit has no effect on the rates of transfer of a phosphate group to HPr. Therefore, some rate constants were considered to be identical:

$$\begin{aligned} kh_1 &= kh_2 \\ kh_7 &= kh_4 \\ kh_8 &= kh_4 \\ kh_9 &= kh_5 \\ kh_{11} &= kh_{10} \\ kh_{13} &= kh_{10} \\ kh_{15} &= kh_{10} \\ kh_{14} &= kh_{12} \\ kh_{18} &= kh_{16} \end{aligned} \qquad \text{(S.28)}$$

According to the assumption (2) mentioned in the main text above, some kinetic constants similarly depend on temperature. Thus, the presence in some cases of Pyr at one of the subunits has no impact on temperature dependence of reactions observed at the other subunit:

$$\begin{aligned}\Delta H_{29} &= \Delta H_{30} \\ \Delta H_{15} &= \Delta H_{16} \\ \Delta H_{20} &= \Delta H_{27} \\ \Delta H_{6} &= \Delta H_{9}\end{aligned} \qquad \text{(S.29.1)}$$

Some activation energies were also equated:

$$\begin{aligned}Ea_8 &= Ea_7 \\ Ea_{11} &= Ea_3 \\ Ea_{15} &= Ea_{16} \\ Ea_{20} &= Ea_{27} \\ Ea_{44} &= Ea_{42} \\ Ea_{41} &= Ea_{40} \\ Ea_{53} &= Ea_{47} = Ea_{54} = Ea_{48} \\ Ea_{50} &= Ea_{51} \\ Ea_{55} &= Ea_{49}\end{aligned} \qquad \text{(S.29.2)}$$

Dissociation constants of proton from catalytic site depend on temperature equally for all the states of subunits:

$$\begin{aligned}\Delta H(K_{0pep}^{h0}) = \Delta H(K_{p0}^{h0}) = \Delta H(K_{ppep}^{h0}) = \Delta H(K_{00}^{h0}) \\ \Delta H(K_{0pep}^{h1}) = \Delta H(K_{p0}^{h1}) = \Delta H(K_{ppep}^{h1}) = \Delta H(K_{00}^{h1})\end{aligned} \qquad \text{(S.30)}$$

The constants of dissociation of proton from the site responsible for dimerization depend similarly on temperature, irrespective of the state of the enzyme (a dimer or a monomer)

$$\Delta H(K_{c}^{h0}) = \Delta H(K_{cd}^{h0})$$

$$\Delta H(K_{c}^{h1}) = \Delta H(K_{cd}^{h1}) \qquad \text{(S.31)}$$

Supplementary material 3.

Expressions for the dependences on pH. In this case, to simplify the description, a number of extra suppositions has been made: (a) protonation of the dimer subunits occurs independently (i.e. there is no co-operativity of subunit protonation); (b) Pyr binding to the enzyme does not affect protonation, and protonation does not affect the binding of Pyr; (c) there are two sites of hydrogen binding per subunit, the protonation of one of the sites influences dimerization, and the protonation of the other site (catalytic) has an effect on all the other reactions; (d) protonation of the site affecting the dimerization occurs independently of the protonation of the catalytic site; (e) constants of proton dissociation from the site affecting the dimerization are different for the monomer and the dimer, and constants of proton dissociation from the catalytic site are identical for the monomer and the dimer. The reactions of the phosphotransfer to HPr were considered as unaffected by the pH value. According to these assumptions, the concentration of the active form of any of the monomer states of the enzyme can be written as follows:

$$E_{xz} = E_{xz}^{t} \cdot \Delta_{xz} \qquad \text{(S.32)}$$

Here, E_{xz}^{t} is the total concentration of a monomer form in one of the states shown in Fig. 1, E_{xz} is the concentration of the catalytically active form of the enzyme in this state, and Δ_{xz} is the function of pH:

$$\Delta_{xz} = \frac{1}{\frac{K_{xz}^{h0}}{h} + 1 + \frac{h}{K_{xz}^{h1}}} \qquad \text{(S.33)}$$

Here, h is the concentration of protons, K_{xz}^{h0} is the dissociation constant, which describes the dissociation of a proton from a singly-protonated site. In this case the state of a monomer is described by two indices x and z. The index "x" is equal to either "p" or "0", implying that the site is phosphorylated or not. The index "z" is equal to either "pep" or "0", implying that PEP is bound to enzyme or not. There is no index for Pyr, since this was assumed to be irrelevant in this

respect. Parameter K_{xz}^{h1} is the dissociation constant which describes the dissociation of a proton from a doubly-protonated site, while symbols “*x*” and “*z*” have the same meaning as mentioned above.

Due to the assumption (a), the concentration of the active form of a dimer at some state can be expressed by the following:

$$D_{xzx1z1} = D_{xzx1z1}^{t} \cdot \Delta_{x1z1} \cdot \Delta_{xz} \qquad \text{(S.34)}$$

Here D_{xzx1z1}^{t} is the concentration of the dimeric form in this state, D_{xzx1z1} is the concentration of the catalytically active form of the enzyme in this state, and Δ_{x1z1} and Δ_{xz} are the functions of pH, according to the expression (14). The state of a dimer is specified by the indices “*x*”, “*x1*”, “*z*”, and “*z1*”. Indices “*x1*” and “*x*” indicate whether subunits of a dimer are phosphorylated or not, and “*z*” and “*z1*” indicate whether PEP is bound to the catalytic site of each of the subunits.

Thus, the equilibrium constants and the rate constants for elementary i-th stages are described by the functions:

$$K_i\,(T,pH) = K_i\,(T_0)\cdot p_i \cdot f(T,\Delta H_i)\cdot g_1(pH, K_{xz}^{h1}, K_{xz}^{h0}, K_{x1z1}^{h1}, K_{x1z1}^{h0}, K_{x2z2}^{h1}, K_{x2z2}^{h0}, K_{x3z3}^{h1}, K_{x3z3}^{h0})$$
$$k_i(T,pH) = k_i(T_0)\cdot p_i \cdot f(T,Ea_i)\cdot g_2(pH, K_{xz}^{h1}, K_{xz}^{h0}, K_{x1z1}^{h1}, K_{x1z1}^{h0})$$

(S.35)

where $K_i\,(T,pH)$ and $k_i\,(T,pH)$ are respectively the equilibrium constant and the rate constant of the i-th elementary reaction at the temperature T and given pH (a few examples of designations are presented in Fig.1 and the explanation of designations can be found in Supplementary 1), $K_i\,(T_0)$ and $k_i\,(T_0)$ are constant factors which depend on pH, p_i is a statistical weight factor,

$$f(T,\Delta H_i) = \exp(\frac{\Delta H_i \cdot (T_0 - T)}{R \cdot T \cdot T_0}) \qquad \text{(S.36)}$$

$$f(T,Ea_i) = \exp(\frac{Ea_i \cdot (T_0 - T)}{R \cdot T \cdot T_0}) \qquad \text{(S.37)}$$

$g_1(pH, K_{xz}^{h1}, K_{xz}^{h0}, K_{x1z1}^{h1}, K_{x1z1}^{h0}, K_{x2z2}^{h1}, K_{x2z2}^{h0}, K_{x3z3}^{h1}, K_{x3z3}^{h0},)$ and $g_2(pH, K_{xz}^{h1}, K_{xz}^{h0}, K_{x1z1}^{h1}, K_{x1z1}^{h0})$ are the functions of pH, symbols "*x*", "*x1*" describe the phosphorylation state of the subunits in the initial state, "*z*" and "*z1*" describe whether PEP is bound to the catalytic site of each subunit in the initial state, "*x2*", "*x3*" describe the phosphorylation state of the subunits in the final state, "*z2*" and "*z3*" describe whether PEP is bound to the catalytic site of each subunit in the final state, so, that $K_{eq}^{i}(T_0) \cdot g_1(pH, K_{xz}^{h1}, K_{xz}^{h0}, K_{x1z1}^{h1}, K_{x1z1}^{h0}, K_{x2z2}^{h1}, K_{x2z2}^{h0}, K_{x3z3}^{h1}, K_{x3z3}^{h0})$ is the equilibrium constant at the temperature T_0 and given *pH*; $k_i(T_0) \cdot g_2(pH, K_h^i)$ is the rate constant at the temperature T_0 and given *pH*. In fact, all the proton dissociation constants described above depend on the temperature in a similar manner to the equilibrium constants according to the formula (12) of main text. The form of the functions g_1 and g_2 depends on the enzymatic state (an example of deriving the functions, as well as the functions for each of the constants are given below).

Let us demonstrate this by taking one constant as an example. Consider the reaction of PEP binding to EI monomer (reaction number 2).

Since, according to the assumption (0) expressed in part «The dependence on pH and temperature» in the main text, only once protonated form of the enzyme is involved in this reaction, the rate of such reaction is equal to:

$$v_2 = k_2(pH) \cdot (EI_t^f \cdot PEP - EI_t^{PEP} \cdot K_2(pH)) = k_2^0 \cdot (EI^{f-} \cdot PEP - EI^{PEP-} \cdot K_2^0) \qquad \text{(S.38)}$$

where EI_t^f is the total concentration of free monomer, EI_t^{PEP} is the total concentration of a monomers with bound PEP, EI^{f-} is the concentration of singly-protonated monomers, EI^{PEP-} is the concentration of the singly-protonated monomers with bound PEP, but K_2^0 and k_2^0 are the constant of PEP dissociation from singly-protonated monomer which is independent of pH, and the rate constant, respectively.

Total concentration of a monomer is equal to the sum of concentrations of non-protonated, singly-protonated and doubly-protonated forms of a monomer:

$$EI_t^f = EI^f + EI^{f-} + EI^{f2-} = EI^f \cdot (1 + \frac{H}{K_{00}^{h0}} \cdot (1 + \frac{H}{K_{00}^{h1}})) = EI^{f-} \cdot (\frac{K_{00}^{h0}}{H} + 1 + \frac{H}{K_{00}^{h1}}) \qquad \text{(S.39)}$$

Similarly,

$$EI_t^{PEP} = EI^{PEP-} \cdot (\frac{K_{0pep}^{h0}}{H} + 1 + \frac{H}{K_{0pep}^{h1}}) \qquad \text{(S.40)}$$

Express EI^{f-} and EI^{PEP-} from formulae (S.39-40) and substitute into the expression for the rate (S.38):

$$v_2 = k_2^0 \cdot (EI^{f-} \cdot PEP - EI^{PEP-} \cdot K_2^0) = k_2^0 \cdot \left[\frac{EI_t^f}{\frac{K_{00}^{h0}}{H} + 1 + \frac{H}{K_{00}^{h1}}} \cdot PEP - \frac{EI_t^{PEP} \cdot K_2^0}{\frac{K_{0pep}^{h0}}{H} + 1 + \frac{H}{K_{0pep}^{h1}}} \right]$$

Calculate the constant of equilibrium K_2 of the reaction 2 ($v_2 = 0$)

$$K_2 = EI_t^f \cdot PEP / EI_t^{PEP} = K_2^0 \cdot \frac{\frac{K_{00}^{h0}}{H} + 1 + \frac{H}{K_{00}^{h1}}}{\frac{K_{0pep}^{h0}}{H} + 1 + \frac{H}{K_{0pep}^{h1}}} = K_2^0 \cdot \frac{\Delta_0^0}{\Delta_0^{pep}}, \qquad \text{(S.41)}$$

where

$$\Delta_0^0 = \frac{K_{00}^{h0}}{H} + 1 + \frac{H}{K_{00}^{h1}}$$
$$\Delta_0^{pep} = \frac{K_{0pep}^{h0}}{H} + 1 + \frac{H}{K_{0pep}^{h1}} \qquad \text{(S.42)}$$

Analogous expressions were also obtained for the other equilibrium constants.

The rate constants are expressed similarly, but in this case it is necessary to describe only the initial state. For instance,

$$v_{15} = k_{15}^0 \cdot (DPEP^- - \frac{DPPyr^-}{K_{t0}^0}) = k_{15}^0 \cdot \left[\frac{DPEP_t}{\Delta_0^{pep} \cdot \Delta_0^0} - \frac{DPPyr}{\Delta_p^0 \cdot \Delta_0^0 \cdot K_{t0}^0} \right] \qquad \text{(S.43)}$$

Therefore,

$$k_{15} = \frac{k_{15}^0}{\Delta_0^{pep} \cdot \Delta_0^0} \qquad \text{(S.44)}$$

$$\Delta_p^0 = \frac{K_{p0}^{h0}}{H} + 1 + \frac{H}{K_{p0}^{h1}} \tag{S.45}$$

In order to write pH-dependencies for all the constants, write auxiliary functions:

$$\Delta_p^{pep} = \frac{K_{ppep}^{h0}}{H} + 1 + \frac{H}{K_{ppep}^{h1}} \tag{S.46}$$

Functions for description of protonation of the dimerization site

$$\Delta_c = \frac{K_c^{h0}}{H} + 1 + \frac{H}{K_c^{h1}} \tag{S.47.1}$$

$$\Delta_{cd} = \frac{K_{cd}^{h0}}{H} + 1 + \frac{H}{K_{cd}^{h1}} \tag{S.47.2}$$

Now write pH dependencies for kinetic constants. Expressions for equilibrium constants are the as follows:

$$
\begin{aligned}
K_1^{dEI} &= K_1^{dEI0} \cdot \frac{(\Delta_c)^2}{(\Delta_{cd})^2} \\
K_{12} &= K_{12}^0 \cdot \frac{\Delta_0^0}{\Delta_0^{pep}} \\
K_{14} &= K_{14}^0 \cdot \frac{\Delta_0^0}{\Delta_0^{pep}} \\
K_{15} &= K_{15}^0 \cdot \frac{\Delta_p^0}{\Delta_0^{pep}} \\
K_{16} &= K_{16}^0 \cdot \frac{\Delta_p^0}{\Delta_0^{pep}} \\
K_{17} &= K_{17}^0 \cdot \frac{\Delta_p^0}{\Delta_0^{pep}} \\
K_{20} &= K_{20}^0 \cdot \frac{\Delta_p^0}{\Delta_0^{pep}} \\
K_{26} &= K_{26}^0 \cdot \frac{\Delta_0^0}{\Delta_0^{pep}} \\
K_{27} &= K_{27}^0 \cdot \frac{\Delta_p^0}{\Delta_0^{pep}} \\
K_{29} &= K_{29}^0 \cdot \frac{\Delta_p^0}{\Delta_p^{pep}} \\
K_{30} &= K_{30}^0 \cdot \frac{\Delta_p^0}{\Delta_p^{pep}} \\
K_{31} &= K_{31}^0 \cdot \frac{\Delta_p^0}{\Delta_p^{pep}} \\
K_{33} &= K_{33}^0 \cdot \frac{\Delta_p^0}{\Delta_p^{pep}} \\
K_{36} &= K_{36}^0 \cdot \frac{\Delta_p^0}{\Delta_0^{pep}} \\
K_{38} &= K_{38}^0 \cdot \frac{\Delta_p^0}{\Delta_p^{pep}} \\
K_{43} &= K_{43}^0 \cdot \frac{\Delta_p^0}{\Delta_p^{pep}} \\
K_{45}^{dEIP2} &= K_1^{dEIP20} \cdot \frac{(\Delta_c)^2}{(\Delta_{cd})^2} \\
K_{48}^{dEIP} &= K_{48}^{dEIP0} \cdot \frac{(\Delta_c)^2}{(\Delta_{cd})^2}
\end{aligned}
\tag{S.48}
$$

Expressions for the rate constants

$$k_1 = \frac{k_1^0}{(\Delta_c)^2}$$

$$k_3 = \frac{k_3^0}{(\Delta_c)^2}$$

$$k_5 = \frac{k_5^0}{(\Delta_c)^2}$$

$$k_7 = \frac{k_7^0}{(\Delta_c)^2}$$

$$k_8 = \frac{k_8^0}{(\Delta_c)^2}$$

$$k_{11} = \frac{k_{11}^0}{(\Delta_c)^2}$$

$$k_{15} = \frac{k_{15}^0}{\Delta_0^0 \cdot \Delta_0^{pep}}$$

$$k_{16} = \frac{k_{16}^0}{\Delta_0^0 \cdot \Delta_0^{pep}}$$

$$k_{17} = \frac{k_{17}^0}{(\Delta_0^{pep})^2}$$

$$k_{20} = \frac{k_{20}^0}{\Delta_p^0 \cdot \Delta_0^{pep}}$$

$$k_{27} = \frac{k_{27}^0}{\Delta_p^0 \cdot \Delta_0^{pep}}$$

$$k_{36} = \frac{k_{36}^0}{\Delta_p^{pep} \cdot \Delta_0^{pep}}$$

$$k_{39} = \frac{k_{39}^0}{(\Delta_{cd})^2}$$

$$k_{40} = \frac{k_{40}^0}{(\Delta_{cd})^2}$$

$$k_{41} = \frac{k_{41}^0}{(\Delta_{cd})^2}$$

$$k_{42} = \frac{k_{42}^0}{(\Delta_{cd})^2}$$

$$k_{44} = \frac{k_7^0}{(\Delta_{cd})^2}$$

$$k_{45} = \frac{k_{45}^0}{(\Delta_{cd})^2}$$

$$k_{47} = \frac{k_{47}^0}{(\Delta_{cd})^2} \qquad \text{(S.49)}$$

$$k_{48} = \frac{k_{48}^0}{(\Delta_{cd})^2}$$

$$k_{49} = \frac{k_{49}^0}{(\Delta_{cd})^2}$$

$$k_{50} = \frac{k_{50}^0}{(\Delta_{cd})^2}$$

$$k_{51} = \frac{k_{51}^0}{(\Delta_{cd})^2}$$

$$k_{52} = \frac{k_{52}^0}{(\Delta_{cd})^2}$$

$$k_{53} = \frac{k_{53}^0}{(\Delta_{cd})^2}$$

$$k_{54} = \frac{k_{54}^0}{(\Delta_{cd})^2}$$

$$k_{55} = \frac{k_{55}^0}{(\Delta_{cd})^2}$$

Figure legends for Supplementary 4

Figure i. Fitted experimental data from reference [17]. Phosphorylation of the EI without HPr.a)time course at T=27˚C, pH=6.5, concentrations: (■)-EI – 0,77 µM, PEP – 10 µM, (●)EI – 154 µM, PEP – 0,2 µM ; b), c) – Equilibrium concentrations of phospho-EI with different initial concentrations of EI and PEP (shown on axis): b)pH6.5, c)pH=7.5, d) Equilibrium concentrations of phospho-EI with different initial concentrations of Pyr, pH=7, EI – 4.6 µM , PEP – 0.3 µM.

Figure ii. Fitted data from reference [6]. Phosphorylation of the HPr . a)Time course of EI phosphorylation by HPrP, T=25˚C, pH=6.5, initial concentrations EI -50 nM, HPr – 18 nM, HPrP – 31 nM, (■)- HPrP;(●)- EIP ; (b) time course of the HPr phosphorylation in presence of PEP, initial concentrations: EI – 140 nM, PEP - 44 µM, HPr – 1,85 µM;

Figure iii. Fitted data from [5]. Dependence of the initial velocity of the HPr phosphorylation on PEP concentration with different HPr concentrations (in µM): (■) – 40, (●) – 20, (▲) – 10, (♦) – 5, (*) – 3.

Figure iv. Data from reference [17] described without fitting. Equilibrium concentrations with different concentrations of EI and PEP T=27˚C: a) pH=8; b) pH=7.

Supplementary material 4.i

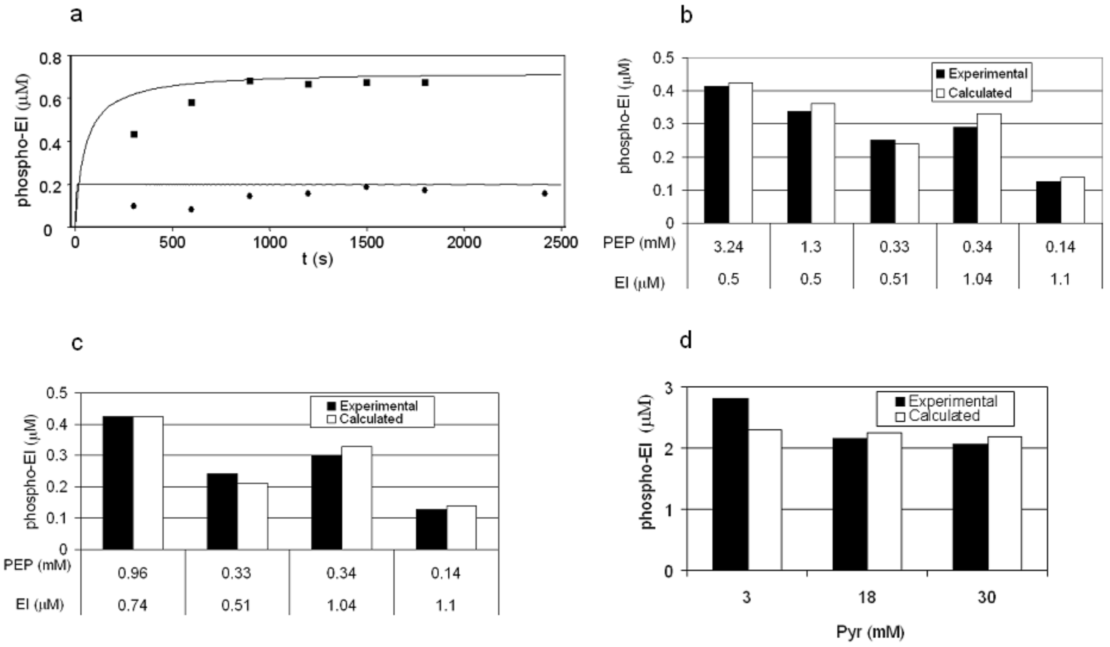

Supplementary material 4.ii

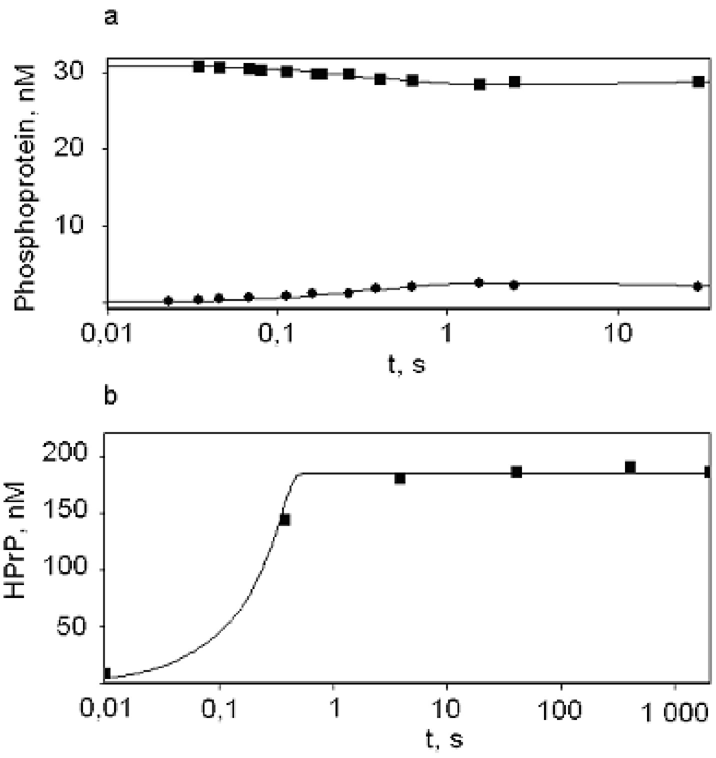

Supplementary material 4.iii

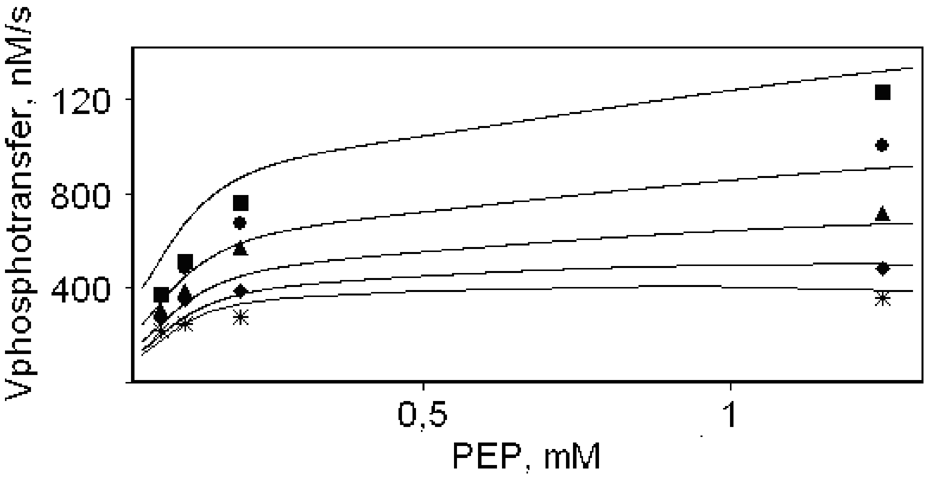

Supplementary material 4.iv

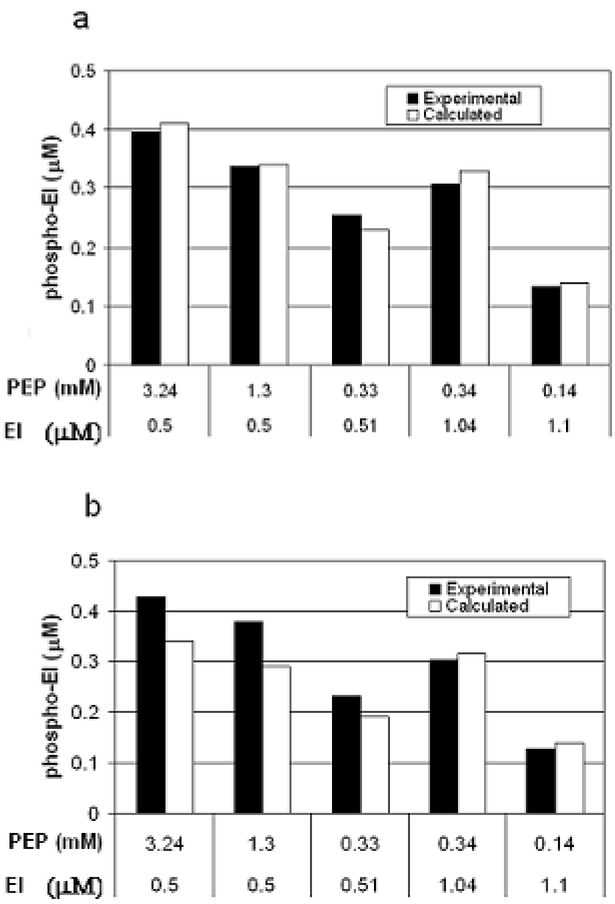

Supplementary material 5.

TABLE S.1. The values of constants obtained by fitting. The constants marked with asterisk are expressed through other constants by the detailed balance ratios (see Text S2). The constants for Pyr dissociation are designated by the letter “R”. other constants by the letter “K”.

Constant designation	Value of constant factor (see eq. (S35 in Text S3))	Value of parameter at T=25˚ C. pH=7.0	Enthalpy [kcal/mol]
The equilibrium constants of elementary processes in the cycle of EI phosphorylation			
K_1^{dEI}	0.001427	$3.811\cdot10^{-4}$ mM	-2.668
K_2	0.07329	$6.658\cdot10^{-1}$ mM	-195.3
$^*K_3^{dEI}$		$7.956\cdot10^{-6}$ mM	
R_4	$8.184\cdot10^{-5}$	$8.184\cdot10^{-5}$ mM	119
$^*K_5^{dEI}$		$9.655\cdot10^{-8}$ mM	
R_6 $(2\cdot\tilde{R}_6)$	$1.414\cdot10^{-1}$	$1.414\cdot10^{-1}$ mM	0
$^*K_7^{dEI}$		$2.089\cdot10^{-4}$ mM	
$^*K_8^{dEI}$		$1.209\cdot10^{-7}$ mM	
R_9 $(\frac{\tilde{R}_9}{2})$	$2.596\cdot10^{-8}$	$2.596\cdot10^{-8}$ mM	0
K_{10}		$4.509\cdot10^{-1}$ mM	
$^*K_{11}^{dEI}$		$8.187\cdot10^{-9}$ mM	
K_{12} $(\frac{\tilde{K}_{12}}{2})$	$1.53\cdot10^{-3}$	$1.39\cdot10^{-2}$ mM	104
R_{13}	$8.421\cdot10^{-8}$	$8.421\cdot10^{-8}$ mM	38.7

K_{14} $(2 \cdot \tilde{K}_{14})$	$8.894 \cdot 10^{-4}$	$8.079 \cdot 10^{-3}$ mM	0
K_{15}^{t0}	2.233	709.9	3.7
K_{16}^{t0}	$1.27 \cdot 10^{-3}$	$4.038 \cdot 10^{-1}$	3.7
K_{17}^{t0} $(2 \cdot \tilde{K}_{17}^{t0})$	60.96	$1.938 \cdot 10^{4}$	-35.5
$^{*}R_{18}$		$4.79 \cdot 10^{-11}$ mM	
$^{*}K_{19}$		$1.683 \cdot 10^{-7}$ mM	
K_{20}^{tp} $(\frac{\tilde{K}_{20}^{tp}}{2})$	$2.816 \cdot 10^{-1}$	89.52	0
$^{*}R_{21}$		$3.343 \cdot 10^{-6}$ mM	
R_{22}	1.831	1.831 mM	0
$^{*}R_{23}$		$3.224 \cdot 10^{-6}$ mM	
$^{*}R_{24}$ $(2 \cdot \tilde{R}_{24})$		$1.226 \cdot 10^{-9}$ mM	
R_{25}	$2.623 \cdot 10^{-5}$	$2.623 \cdot 10^{-5}$ mM	0
K_{26}	$1.05 \cdot 10^{-2}$	$9.558 \cdot 10^{-2}$ mM	0
K_{27}	$1.071 \cdot 10^{-4}$	$3.404 \cdot 10^{-2}$ mM	
R_{28} $(\frac{\tilde{R}_{28}}{2})$	$1.748 \cdot 10^{-5}$	$1.748 \cdot 10^{-5}$ mM	128.5
K_{29}	$2.759 \cdot 10^{-5}$	$5.974 \cdot 10^{-3}$ mM	-70.3
K_{30}	$7.007 \cdot 10^{-4}$	$1.517 \cdot 10^{-1}$ mM	-70.3
K_{31}	$4.126 \cdot 10^{-5}$	$8.933 \cdot 10^{-3}$ mM	0
$^{*}K_{32}$		$9.748 \cdot 10^{-4}$ mM	

K_{33}	$6.394 \cdot 10^{-4}$	$1.384 \cdot 10^{-1}$ mM	189.8
$^{*}R_{34}$		$1.033 \cdot 10^{-6}$ mM	
$^{*}K_{35}$		$5.628 \cdot 10^{-3}$ mM	
K_{36}^{tp}	$9.812 \cdot 10^{-4}$	$3.12 \cdot 10^{-1}$ mM	-0.6
$^{*}R_{37}$		$1.231 \cdot 10^{-7}$ mM	
K_{38} $(2 \cdot \widetilde{K}_{38})$	$2.08 \cdot 10^{-3}$	$4.504 \cdot 10^{-1}$ mM	0.9
$^{*}K_{39}^{dEIP2}$		$7.064 \cdot 10^{-1}$ mM	
$^{*}K_{40}^{dEIP2}$		$4.52 \cdot 10^{-4}$ mM	
$^{*}K_{41}^{dEIP2}$		$7.957 \cdot 10^{-12}$ mM	
$^{*}K_{42}^{dEIP2}$		$1.863 \cdot 10^{-8}$ mM	
K_{43}	$1.331 \cdot 10^{-6}$	$2.882 \cdot 10^{-4}$ mM	0
$^{*}K_{44}^{dEIP2}$		$2.589 \cdot 10^{-4}$ mM	
K_{45}^{dEIP2}	$3.523 \cdot 10^{-6}$	$9.409 \cdot 10^{-7}$ mM	15.7
R_{46}	$8.825 \cdot 10^{-4}$	$8.825 \cdot 10^{-4}$ mM	2.787
$^{*}K_{47}^{dEIP}$		$1.653 \cdot 10^{-2}$ mM	
K_{48}^{dEIP}	$2.983 \cdot 10^{-5}$	$7.969 \cdot 10^{-6}$ mM	0
$^{*}K_{49}^{dEIP}$		$4.195 \cdot 10^{-3}$ mM	
$^{*}K_{50}^{dEIP}$		$4.18 \cdot 10^{-9}$ mM	
$^{*}K_{51}^{dEIP}$		$1.144 \cdot 10^{-6}$ mM	
$^{*}K_{52}^{dEIP}$		$3.546 \cdot 10^{-5}$ mM	

$^{*}K_{53}^{dEIP}$		$9.677\cdot10^{-9}$ mM	
$^{*}K_{54}^{dEIP}$		$2.554\cdot10^{-6}$ mM	
$^{*}K_{55}^{dEIP}$		$5.294\cdot10^{-5}$ mM	
The rate constants of the elementary processes in the cycle of EI phosphorylation			
	Value of constant factor (see (16))	Value of parameter at T=25° C. pH=7.0	Activation energy (kcal/mole)
k_1	65.96	38.12 $mM^{-1}\cdot s^{-1}$	0
k_3	$5.943\cdot10^{-10}$	$2.718\cdot10^{-10}$ $mM^{-1}\cdot s^{-1}$	54.3
k_5	70.02	38.44 $mM^{-1}\cdot s^{-1}$	0
k_7	$2.49\cdot10^{-1}$	$2.103\cdot10^{-1}$ $mM^{-1}\cdot s^{-1}$	0
k_8	1.546	$8.488\cdot10^{-4}$ $mM^{-1}\cdot s^{-1}$	0
k_{11}	$4.944\cdot10^{-2}$	$1.938\cdot10^{-2}$ $mM^{-1}\cdot s^{-1}$	54.3
k_{15}	$1.307\cdot10^{-5}$	$3.594\cdot10^{3}$ s^{-1}	193
k_{16}	$7.129\cdot10^{4}$	$1.587\cdot10^{3}$ s^{-1}	193
$k_{17}\cdot$ $(2\cdot\tilde{k}_{17})$	$1.287\cdot10^{3}$	281.1 s^{-1}	0
k_{20}	$1.217\cdot10^{4}$	8.1 s^{-1}	0
k_{27}	$2.888\cdot10^{7}$	$1.923\cdot10^{4}$ s^{-1}	0
k_{36}	$2.552\cdot10^{-3}$	$1.97\cdot10^{-6}$ s^{-1}	0
k_{39}	1.017	$1.481\cdot10^{-1}$ $mM\cdot s^{-1}$	96.6
k_{40}	0	0	0
k_{41}	0	0	0
k_{42}	0	0	0

k_{44}	0	0	0
k_{45}	$2.319\cdot10^{-2}$	$3.376\cdot10^{-3}$ mM·s^{-1}	0
k_{47}	0	0	213
k_{48}	$6.06\cdot10^{-2}$	$8.823\cdot10^{-3}$ mM·s^{-1}	213
k_{49}	0	0	0
k_{50}	0	0	0
k_{51}	0	0	0
k_{52}	$3.01\cdot10^{-2}$	$4.382\cdot10^{-3}$ mM·s^{-1}	6.7
k_{53}	$8.858\cdot10^{-2}$	$1.29\cdot10^{-2}$ mM·s^{-1}	213
k_{54}	0	0	213
k_{55}	104.1	15.15 mM·s^{-1}	0
The constants of proton dissociation			
	Value of constant factor (see (16))	Value of parameter at T=25˚ C. pH=7.0	Enthalpy (kcal/mol)
K_{00}^{h0}	$6.344\cdot10^{-6}$	$6.344\cdot10^{-6}$ mM	6.6
K_{00}^{h1}	$5.409\cdot10^{-6}$	$5.409\cdot10^{-6}$ mM	0
K_{0pep}^{h0}	$1.345\cdot10^{-6}$	$1.345\cdot10^{-6}$ mM	6.6
K_{0pep}^{h1}	$8.782\cdot10^{-5}$	$8.782\cdot10^{-5}$ mM	0
K_{p0}^{h0}	$2.806\cdot10^{-6}$	$2.806\cdot10^{-6}$ mM	6.6
K_{p0}^{h1}	$1.464\cdot10^{-7}$	$2.806\cdot10^{-6}$ mM	0
K_{ppep}^{h0}	$5.982\cdot10^{-7}$	$5.982\cdot10^{-7}$ mM	6.6

K_{ppep}^{h1}	$4.642 \cdot 10^{-5}$	$4.642 \cdot 10^{-5}$ mM	0
K_{cd}^{h0}	$1.884 \cdot 10^{-8}$	$1.884 \cdot 10^{-8}$ mM	3.2
K_{cd}^{h1}	$6.17 \cdot 10^{-5}$	$6.17 \cdot 10^{-5}$ mM	0
K_{c}^{h0}	$4.859 \cdot 10^{-7}$	$4.859 \cdot 10^{-7}$ mM	3.2
K_{c}^{h1}	$2.86 \cdot 10^{-4}$	$2.86 \cdot 10^{-4}$ mM	
The equilibrium constants of HPr phosphorylation			
	Value of constant factor (see (16))	Value of constant at T=25˚ C. pH=7.0	Enthalpy (kcal/mole)
$^{*}Kh_{1}$		231.6	
Kh_{2}	21.48	21.48	0
$^{*}Kh_{3}$		$9.296 \cdot 10^{-3}$	
Kh_{4}^{d}	$5.738 \cdot 10^{-2}$	$5.738 \cdot 10^{-2}$	0
$^{*}Kh_{5}^{d}$		$8.311 \cdot 10^{-2}$	
$^{*}Kh_{5'}^{d}$		$5.237 \cdot 10^{-2}$	
$^{*}Kh_{6}^{d}$		4.191	
$^{*}Kh_{7}^{d}$		$3.823 \cdot 10^{-2}$	
$^{*}Kh_{7'}^{d}$		$5.478 \cdot 10^{-7}$	
$^{*}Kh_{8}^{d}$		$1.402 \cdot 10^{-5}$	
$^{*}Kh_{9}^{d}$		$3.172 \cdot 10^{-3}$	
$^{*}Kh_{9'}^{d}$		$6.238 \cdot 10^{-3}$	

Kh_{10}^{d1}	84.34	84.34	13.3
$^{*}Kh_{11}^{d1}$		$8.523 \cdot 10^{4}$	
$^{*}Kh_{12}^{d1}$		580	
$^{*}Kh_{13}^{d1}$		$5.948 \cdot 10^{9}$	
$^{*}Kh_{14}^{d1}$		$2.221 \cdot 10^{4}$	
$^{*}Kh_{15}^{d1}$		2.015	
$^{*}Kh_{16}^{d1}$		920.5	
$^{*}Kh_{17}^{d1}$		641.3	
$^{*}Kh_{18}^{d1}$		$1.129 \cdot 10^{4}$	
The rate constants of HPr phosphorylation			
	Value of constant factor (see (16))	Value of constant at T=25˚ C. pH=7.0	Activation energy (kcal/mole)
$^{*}kh_{1}$		$9.673 \cdot 10^{4}\ s^{-1}$	
kh_{2}	9.673E4	$9.673 \cdot 10^{4}\ s^{-1}$	0
kh_{3}	1.718E-7	$1.718 \cdot 10^{-7}\ s^{-1}$	0
$kh_{4} \cdot\ (2 \cdot \tilde{k}h_{4})$	$9.512 \cdot 10^{-3}$	$9.512 \cdot 10^{-3}\ s^{-1}$	0
kh_{5}	0	0	0
$kh_{6} \cdot\ (2 \cdot \tilde{k}h_{6})$	$1.595 \cdot 10^{-1}$	$1.595 \cdot 10^{-1}\ s^{-1}$	0.03
$^{*}kh_{7}$		$9.512 \cdot 10^{-3}\ s^{-1}$	
$^{*}kh_{8}$		$9.512 \cdot 10^{-3}\ s^{-1}$	
$^{*}kh_{9}$	0	0	

kh_{10}	$1.578699 \cdot 10^{6}$	$1.579 \cdot 10^{6}$ s^{-1}	0
$^{*}kh_{11}$		$1.579 \cdot 10^{6}$ s^{-1}	
kh_{12}	0	0	0
$^{*}kh_{13}$		$1.579 \cdot 10^{6}$ s^{-1}	
$^{*}kh_{14}$	0	0	
$^{*}kh_{15}$		$1.579 \cdot 10^{6}$ s^{-1}	
kh_{16}	$4.069896 \cdot 10^{6}$	$4.07 \cdot 10^{6}$ s^{-1}	0
kh_{17}	0	0	0
$^{*}kh_{18}$		$4.07 \cdot 10^{6}$ s^{-1}	0
HPrP hydrolysis			
	Value of constant factor (see (16))	Value of constant at T=25˚ C. pH=7.0	Enthalpy (activation energy) (kcal/mol)
khyd	$1.024 \cdot 10^{-5}$	$1.023740 \cdot 10^{-5}$ $mM \cdot s^{-1}$	0
K_{hyd}	4.553	3.9588 mM	0
